# Proteomic analysis of glycosomes from *Trypanosoma cruzi* epimastigotes

**DOI:** 10.1016/j.molbiopara.2019.02.008

**Published:** 2019-04

**Authors:** Héctor Acosta, Richard Burchmore, Christina Naula, Melisa Gualdrón-López, Ender Quintero-Troconis, Ana J. Cáceres, Paul A.M. Michels, Juan Luis Concepción, Wilfredo Quiñones

**Affiliations:** a Laboratorio de Enzimología de Parásitos, Facultad de Ciencias, Universidad de Los Andes, Mérida, 5101, Venezuela; b Institute of Infection, Immunity and Inflammation, College of Medical, Veterinary and Life Sciences, University of Glasgow, Glasgow, G12 8QQ, UK; c Instituto Salud Global, Hospital Clinic-Universitat de Barcelona, and Institute for Health Sciences Trias i Pujol, Barcelona, Spain; d Centre for Immunity, Infection and Evolution and Centre for Translational and Chemical Biology, The University of Edinburgh, Edinburgh, EH9 3FL, UK

**Keywords:** ACBP, acyl-CoA-binding protein, ADH, alcohol dehydrogenase, APRT, adenine phosphoribosyltransferase, DHAP, dihydroxyacetone phosphate, FBPase, fructose-1,6-bisphosphatase, FH, fumarate hydratase, FRD, fumarate reductase, GALK, galactokinase, GAPDH, glyceraldehyde-3-phosphate dehydrogenase, GAT, glycosomal ABC transporter, GDH, glutamate dehydrogenase, G6P, glucose 6-phosphate, Glyc3P, glycerol 3-phosphate, GPDH, glycerol-3-phosphate dehydrogenase, HADH, hydroxyacid dehydrogenase, HGPRT, hypo-xanthine-guanine phosphoribosyltransferase, HK, hexokinase, HMG-CoA, 3-hydroxy-3-methyl-glutaryl Coenzyme A, IMPDH, inosine-5′-monophosphate dehydrogenase, MCF, mitochondrial carrier family, MDH, malate dehydrogenase, PAS, Per-ARNT-Sim, PEP, phosphoenolpyruvate, PEPCK, phosphoenolpyruvate carboxykinase, PEX, peroxin, PFK, phosphofructokinase, 3PGA, 3-phosphoglycerate, PGK, phosphoglycerate kinase, PMP, peroxisomal membrane protein, PPi, inorganic pyrophosphate, PPDK, pyruvate phosphate dikinase, PPP, pentose-phosphate pathway, PTS, peroxisomal-targeting signal, SOD, superoxide dismutase, *Trypanosoma cruzi*, Glycosome, Glycolysis, Metabolism, Proteomics, Cell fractionation, Mass spectrometry

## Abstract

In *Trypanosoma cruzi*, the causal agent of Chagas disease, the first seven steps of glycolysis are compartmentalized in glycosomes, which are authentic but specialized peroxisomes. Besides glycolysis, activity of enzymes of other metabolic processes have been reported to be present in glycosomes, such as β-oxidation of fatty acids, purine salvage, pentose-phosphate pathway, gluconeogenesis and biosynthesis of ether-lipids, isoprenoids, sterols and pyrimidines. In this study, we have purified glycosomes from *T. cruzi* epimastigotes, collected the soluble and membrane fractions of these organelles, and separated peripheral and integral membrane proteins by Na_2_CO_3_ treatment and osmotic shock. Proteomic analysis was performed on each of these fractions, allowing us to confirm the presence of enzymes involved in various metabolic pathways as well as identify new components of this parasite’s glycosomes.

## Highlights

• New metabolic routes and membrane proteins were identified in the organelles.
• Exponential and stationary growth-phase cells vary little in glycosomal proteins.
• A variety of proteins bearing a typical PTS1 but with unknown function were found.

## Graphical abstract

**Figure:**
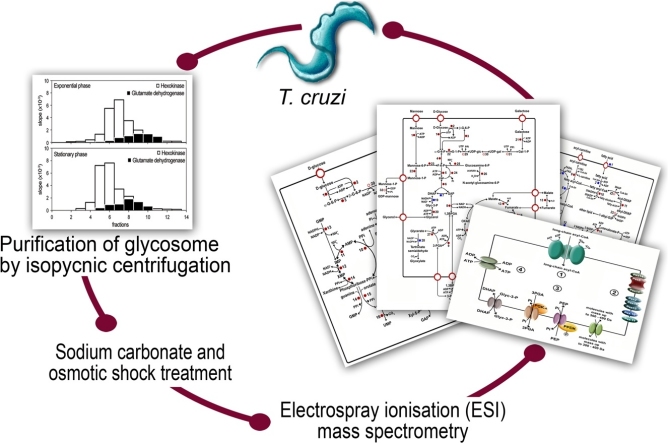

## 1 Introduction

Whereas in almost all eukaryotic organisms glycolysis is a process that occurs in the cytosol, in Kinetoplastea the major part of the pathway is localized in organelles called glycosomes. The glycosomes of *T. cruzi* contain the enzymes converting glucose into 3-phosphoglycerate (3PGA); only the last three enzymes of the glycolytic pathway are present in the cytosol [1,2]. A consequence of this organization is that, inside these organelles, the ATP consumed by hexokinase (HK) and phosphofructokinase (PFK) is regenerated by a phosphoglycerate kinase (PGK) and, in two auxiliary branches of glycolysis, a phospho*enol*pyruvate carboxykinase (PEPCK) and/or pyruvate phosphate dikinase (PPDK). The regeneration of ATP by PEPCK and/or PPDK implies that entry of cytosolic PEP is absolutely necessary. Similarly, the NADH formed in the reaction catalyzed by glyceraldehyde-3-phosphate dehydrogenase (GAPDH) is re-oxidized inside the organelles by reduction of oxalocetate (produced from PEP by PEPCK) to malate by a glycosomal malate dehydrogenase (MDH) and in a subsequent reduction of fumarate produced from the malate to succinate by a soluble NADH-dependent fumarate reductase (FRD) [3]. Another route that may contribute to the regeneration of glycosomal NAD^+^ involves a NAD-dependent glycerol-3-phosphate dehydrogenase (GPDH) [4] that catalyzes the reduction of dihydroxyacetone phosphate (DHAP) to glycerol 3-phosphate (Glyc3P). Subsequently, the electrons are transferred to oxygen *via* a mitochondrial electron-transport system and a redox shuttle comprising a putative transporter in the glycosomal membrane which exchanges Glyc3P for DHAP. The transporter remains to be identified, but its existence is inferred from the requirement for strict coupling of the fluxes by which the two triosephosphates are exchanged. All glycosomal enzymes present high latency upon isolation of the organelles, which is in accordance with the notion that the glycosomal membrane acts as a permeability barrier for the enzymes and their cofactors, whereas it, like the membrane of peroxisomes in other organisms, may allow passage of small metabolites through channels [[5], [6], [7]]. In addition, another important pathway of glucose oxidation present in glycosomes (as well as the cytosol) is the pentose-phosphate pathway (PPP). The PPP usually has two major roles, namely the reduction of NADP^+^ to NADPH, as well as the production of ribose 5-phosphate to be used as substrate for the synthesis of a variety of cell components [8].

Besides glycolysis and the PPP, the presence of enzymes involved in other metabolic routes has also been described for glycosomes of different trypanosomatids (*T. cruzi, Trypanosoma brucei, Leishmania* spp.): gluconeogenesis, purine salvage, β-oxidation of fatty acids and biosynthesis of ether-lipids, isoprenoids, sterols and pyrimidines [[9], [10], [11], [12], [13], [14]].

It has been observed that several of the enzymes belonging to these pathways are essential for the survival of the bloodstream form of *T. brucei* [[15], [16], [17]]. Therefore, some of these glycosomal enzymes are considered as potential pharmacological targets [14,[18], [19], [20]]. Glycosomes, like peroxisomes contain peroxins (PEX proteins), proteins involved in the different routes of the biogenesis of the organelles, such as the import of proteins synthesized in the cytosol into the matrix of the organelles [20]. Many, but not all, of the glycosomal matrix proteins exhibit a peroxisomal-targeting signal (PTS) comprising a partially conserved motif at their C-terminus or close to their N-terminal end, called PTS1 or PTS2, respectively [17,19,21,22].

In the study reported in this paper, we have purified glycosomes from *T. cruzi* and separated the soluble matrix from the membranes. Subsequently, the proteins attached peripherally to the membrane were separated from the integral membrane proteins through treatment with Na_2_CO_3_ and exposure to an osmotic shock [23]. To obtain information about the growth-dependent variation of the glycosomal membrane and soluble proteins, we have chosen to compare glycosomes from exponentially growing epimastigotes, whose metabolism is essentially glycolytic, with those from stationary-phase epimastigotes, in which the metabolism has shifted to catabolism of amino acids as their carbon and energy source [24,25].

## 2 Materials and methods

### 2.1 Parasites culture

*Trypanosoma cruzi* epimastigotes (EP strain) were axenically cultivated in liver infusion-tryptose (LIT) medium at 28 °C as previously described [26]. Cells were harvested when they reached the mid-exponential and stationary growth phase at OD_600 nm_ values of 0.6 and 1.2, respectively.

### 2.2 Glycosome purification

The parasites (exponential and stationary phase) were centrifuged at 800 x g for 10 min at 4 °C, and washed twice with isotonic buffer A (20 mM Tris−HCl, pH 7.2 with 225 mM sucrose, 20 mM KCl, 10 mM KH_2_PO_4_, 5 mM MgCl_2_, 1 mM Na_2_EDTA) and hypotonic buffer B (25 mM Tris−HCl, pH 7.6 with 250 mM sucrose, 25 mM NaCl, 1 mM Na_2_EDTA). Homogenates of exponential and stationary phase parasites were obtained by grinding washed cells with silicon carbide (200 mesh) in the presence of a 1/100 (v/v) protease inhibitors cocktail (final concentrations: 1 μM pepstatin, 0.16 μM antipain, 5 μM leupeptin, 100 μM tosyl leucine chloromethyl ketone, 2.8 μM trans-epoxysuccinyl-L-leucylamido-(4-guanidino) butane (E-64), 100 μM sodium ethylene diamine tetraacetate, 500 μM phenylmethyl sulfonylfluoride). Parasite disruption was checked for being at least 90% complete by light microscopy. The homogenates were centrifuged first for 10 min at 1000 x g at 4 °C in order to remove silicon carbide, nuclei and intact cells. The resulting supernatant was centrifuged for 20 min at 5000×*g* at 4 °C to remove a large granular fraction as a pellet. A small granular pellet (glycosome-enriched fraction) was obtained after a centrifugation step at 3000×*g* for 20 min at 4 °C. This pellet was resuspended in 1.5 ml of buffer B containing a protease inhibitors cocktail and loaded on top of a 35 ml linear 0.25–2.5 M sucrose gradient. Centrifugation was performed for 2 h at 170,000×*g* and 4 °C using a vertical rotor (Sorvall TV-860). The glycosome-enriched fraction (1.21–1.25 *g*. cm^−3^) was applied to a second sucrose gradient. Fractions of 1.9 ml were collected from the bottom of the tube after puncture [23,27].

### 2.3 Isolation of glycosomal membrane proteins from T. cruzi epimastigotes by sodium carbonate and osmotic shock treatment of the organelles

For the sodium carbonate or osmotic shock treatment, one volume of the glycosomal fractions, with protein concentrations of 7 mg. ml^−1^ and 5 mg. ml^−1^ for the samples derived from the exponential and stationary growth phase, respectively, was mixed with about 100 volumes of 100 mM cold sodium carbonate (pH 11.5) or with cold milliQ water according to [23,28] and incubated at 0 °C for 30 min before ultracentrifugation at 105,000×*g* for 2 h at 4 °C. The pellet was homogenized in milliQ water with a potter and further centrifuged at 105,000×*g* for 2 h at 4 °C. This procedure allowed the separation of matrix proteins and detached peripheral membrane proteins in the supernatant and a membrane fraction with integral proteins in the pellet after the sodium carbonate treatment and peripheral and integral proteins in the pellet after the osmotic shock treatment. The protein obtained in supernatants of sodium carbonate and osmotic shock treatment were lyophilized in a Labconco freeze dryer and stored at –80 °C until its later use.

### 2.4 Mass spectrometry analysis

Proteins were digested with trypsin using the FASP protocol as described by [29]. Peptides were solubilized in 2% acetonitrile with 0.1% trifluoro acetic acid and fractionated on a nanoflow uHPLC system (Thermo ScientificRSLCnano) before online analysis by electrospray ionisation (ESI) mass spectrometry on an Orbitrap Elite MS (Thermo Scientific). Peptide separation was performed on a Pepmap C18 reversed phase column (Thermo Scientific). Peptides were desalted and concentrated for 4 min on a C18 trap column followed by an acetonitrile gradient (in 0.1% v/v formic acid) (3.2–32% v/v 4–27 min, 32% to 80% v/v 27–36 min, held at 80% v/v 36–41 min and re-equilibrium at 3.2%) for a total time of 45 min. A fixed solvent flow rate of 0.3 μl. min^−1^ was used for the analytical column. The trap column solvent flow was 25 μl. min^−1^ of 2% acetonitrile with 0.1% v/v trifluoroacetic acid. Eluate was delivered online to the Orbitrap Elite MS (Thermo Scientific), acquiring a continuous duty cycle of a high resolution precursor scan at 60,000 RP (at 400 *m/z*), while simultaneously acquiring the top 20 precursors subjected to CID fragmentation in the linear ion trap. Singly charged ions were excluded from selection, while selected precursors are added to a dynamic exclusion list for 120 s. Protein identifications were assigned using the Mascot search engine (v2.5.1) to interrogate *T. cruzi* CL Brener protein coding sequences in the NCBI database, allowing a mass tolerance of 10 ppm for the precursor and 0.6 Da MS/MS matching.

### 2.5 Bioinformatic analysis

To classify type-1 and type-2 peroxisomal targeting signals in the glycosomal proteins revealed in this study, the [ASCGPNYTV][KNRHQDS][LMVAIF] motif was used to recognize proteins having the PTS1 sequence, while the [RKHQ][VLIWFY]X5[HKQR][ILVYAF] sequence was used to identify proteins with the PTS2 sequence [30]. PTS1 and PTS2 containing sequences identified in our analysis were searched in the TriTryp and GeneDB databases. A comparison was made of the protein repertoires found in the glycosomes of epimastigotes grown to exponential and stationary phases.

### 2.6 Protein determination

Protein concentration was determined by the method of [31] using the Bio-Rad Bradford protein assay.

### 2.7 Enzymatic assays

Hexokinase (HK) and glutamate dehydrogenase (GDH) activities were assayed spectrophotometrically according to [32] and [33], respectively. The activities were measured in a Hewlett-Packard 8452 diode array spectrophotometer at 340 nm at 28 °C, in the presence of 0.1% (v/v) Triton X-100 and 150 mM NaCl in order to solubilize membrane and eliminate latency.

## 3 Results and discussion

### 3.1 Glycosome purification and proteome analysis by mass spectrometry

Glycosomes were isolated from epimastigotes in exponential and stationary growth phase by differential centrifugation followed by two rounds of isopycnic ultracentrifugation on a sucrose gradient. Figure SI (Supplementary Information) shows that most glycosomes were recovered in fractions corresponding to densities of 1.23 to 1.24 *g*. cm^−3^ (fractions 6 and 7) after the isopycnic ultracentrifugation (HK activity was used as glycosomal marker). In contrast, most GDH, a mitochondrial marker enzyme, was recovered in fractions corresponding to densities of 1.15 to 1.17 *g*. cm^-3^ (fractions 8–10). A small overlap of the HK activity with that of GDH (in fractions 8–10) was observed, which could be due to rupture of glycosomes during isolation. Analysis of the HK and GDH activities showed that fractions 3 to 6 contained about 35% of the total HK activity measured, and only 5% of the total GDH activity. For this reason, we decided to use fractions 3 to 6 for glycosomal membrane protein isolation by sodium carbonate and osmotic shock treatment. The isolation procedures and the mass spectrometry data acquisition using the samples are described in Materials and methods, sections 2.3 and 2.4.

Studies with peroxisomes demonstrated the involvement of vesicles derived from mitochondria as well as the endoplasmic reticulum in the biogenesis of the organelles, implying that the classification of some proteins as "contaminants" in a peroxisomal preparation must be carefully considered [[34], [35], [36]]. In this respect, we used three criteria to assign proteins as authentic to glycosomes: (i) the results of our proteomic analysis; (ii) the presence of a glycosomal import signal of type PTS1 or PTS2; and (iii) previous publications about the association of proteins with *T. cruzi* glycosomes and a comparison with the data reported in proteomics studies of glycosomes from other kinetoplastids [22,37,38]. For this last criterion, special emphasis was placed on the work of [22] with the organelles of procyclic *T. brucei*, because this latter study can be considered as presenting a minimal level of contamination as a result of the powerful epitope-tagged method of glycosome isolation used.

### 3.2 Glycolysis

**Fig. 1:**
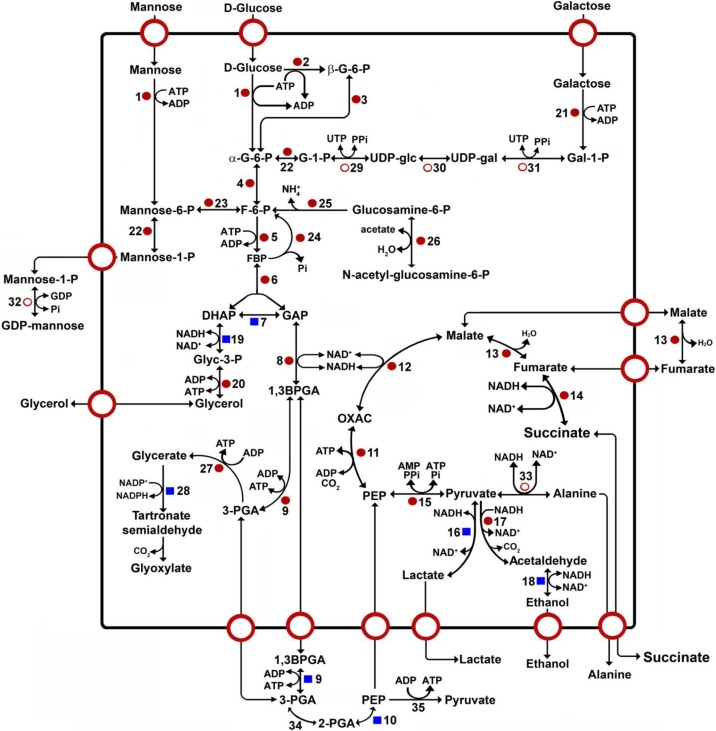
Carbohydrate metabolism. ●: Proteins found in the mass analysis that present a PTS1 or PTS2 motif; ■: Proteins found in the mass analysis but which do not present a PTS1 or PTS2 sequence; ○: Proteins not detected in the mass analysis but reported in literature. 1. hexokinase, 2. glucokinase-1, 3. D-hexose-6-phosphate-1-epimerase, 4. glucose-6-phosphate isomerase, 5. 6-phospho-1-fructokinase, 6. fructose-bisphosphate aldolase, 7. triosephosphate isomerase, 8. glyceraldehyde-3-phosphate dehydrogenase, 9. phosphoglycerate kinase, 10. enolase, 11. phospho*enol*pyruvate carboxykinase, 12. glycosomal malate dehydrogenase, 13. fumarate hydratase, 14. NADH-dependent fumarate reductase, 15. pyruvate phosphate dikinase, 16. D-isomer specific 2-hydroxyacid dehydrogenase-protein, 17. aldehyde dehydrogenase, 18. alcohol dehydrogenase, 19. glycerol-3-phosphate dehydrogenase, 20. glycerol kinase, 21. galactokinase, 22. phosphomannomutase-like protein, 23. phosphomannose isomerase, 24. fructose-1,6-bisphosphatase, 25. glucosamine-6-phosphate isomerase, 26. N-acetylglucosamine-6-phosphate deacetylase-like protein, 27. glycerate kinase (hypothetical protein), 28. 2-hydroxy-3-oxopropionate reductase, 29. UTP-glucose-1-phosphate uridylyltransferase [22], 30. UDP-galactose 4-epimerase [21,125], 31. UDP-sugar pyrophosphorylase [126], 32. GDP-mannose pyrophosphorylase [127], 33. alanine dehydrogenase [12], 34. phosphoglycerate mutase [2], 35, pyruvate kinase [2]. G-6-P: glucose 6-phosphate, G-1-P: glucose 1-phosphate, UDP-glc: UDP-glucose, UDP-gal: UDP-galactose, Gal-1-P: galactose 1-phosphate, GDP-gal: GDP-galactose, Mannose-1-P: mannose 1-phosphate, Mannose-6-P: mannose 6-phosphate, F-6-P: fructose 6-phosphate, FBP: fructose 1,6-bisphosphate, DHAP: dihydroxyacetone phosphate, GAP: glyceraldehyde 3-phosphate, Glyc-3-P: glycerol 3-phosphate, 1,3BPGA: 1,3-bisphosphoglycerate, 3-PGA: 3-phosphoglycerate, 2-PGA: 2-phosphoglycerate, PEP: phospho*enol*pyruvate, OXAC: oxaloacetate.

**Fig. 2:**
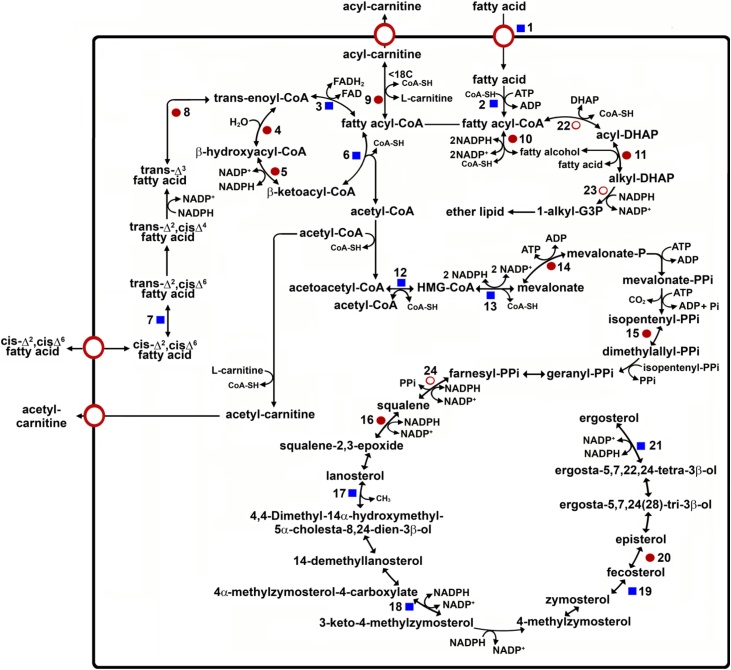
Lipid metabolism. ●: Proteins found in the mass analysis that present a PTS1 or PTS2 motif; ■: Proteins found in the mass analysis but which do not present a PTS1 or PTS2 sequence; ○: Proteins not detected in the mass analysis but reported in literature. 1. ABC transporter GAT1, 2. fatty-acyl CoA synthetase, 3. acyl-CoA dehydrogenase, 4. enoyl-CoA hydratase, 5. 3-hydroxyacyl-CoA dehydrogenase, 6. 3-ketoacyl-CoA thiolase, 7. 3,2-trans-enoyl-CoA isomerase, 8. enoyl-CoA isomerase, 9. carnitine/choline O-acyltransferase, 10. fatty-acyl-CoA reductase, 11. alkyl-dihydroxyacetone phosphate synthase, 12. 3-hydroxy-3-methylglutaryl CoA synthase, 13. 3-hydroxy-3-methylglutaryl-CoA reductase, 14. mevalonate kinase, 15. isopentenyl-diphosphate delta-isomerase, 16. squalene monooxygenase, 17. lanosterol 14-alpha-demethylase, 18. NAD(P)-dependent steroid dehydrogenase protein, 19. sterol 24-C-methyltransferase, 20. C-8 sterol isomerase, 21. sterol C-24 reductase, 22. DHAP acyltransferase [74], 23. 1-alkyl G3P:NADP^+^ oxidoreductase [21,67], 24. squalene synthase [81]. DHAP: dihydroxyacetone phosphate, HMG-CoA: 3-hydroxy-3-methylglutaryl-CoA.

**Fig. 3:**
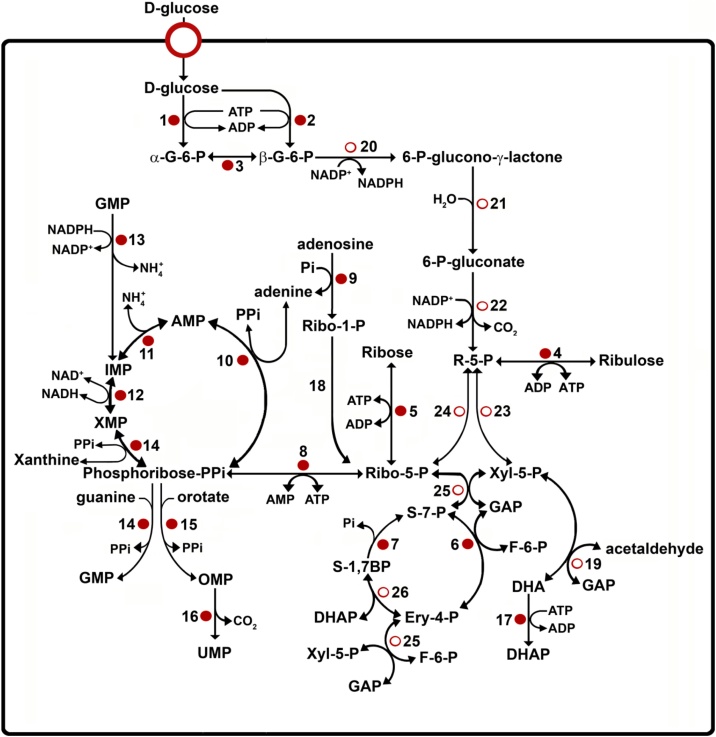
Purine salvage, pyrimidine biosynthesis and pentose-phosphate pathway. ●: Proteins found in the mass analysis that present a PTS1 or PTS2 motif; ○: Proteins not detected in the mass analysis but reported in literature.1. hexokinase, 2. glucokinase 1, 3. D-hexose-6-phosphate-1-epimerase, 4. ribulokinase, 5. ribokinase, 6. transaldolase, 7. sedoheptulose-1,7-bisphosphatase, 8. phosphoribosylpyrophosphate synthetase (hypothetical protein), 9. nucleoside phosphorylase, 10. adenine phosphoribosyltransferase, 11. AMP deaminase, 12. inosine-5′-monophosphate dehydrogenase, 13. guanosine monophosphate reductase, 14. hypoxanthine-guanine phosphoribosyltransferase, 15. orotate phosphoribosyltransferase, 16. orotidine-5-phosphate decarboxylase, 17. dihydroxyacetone kinase, 18. phosphopentomutase, 19. dihydroxyacetone synthase [128], 20. glucose-6-phosphate dehydrogenase [50], 21. lactonase [50], 22. 6-phosphogluconate dehydrogenase [129], 23. ribulose-5-phosphate epimerase [130], 24. ribulose-5-phosphate isomerase [50], 25. transketolase [50]; 26. sedoheptulose-1,7-phosphate transaldolase [21]. G-6-P: glucose 6-phosphate, R-5-P: ribulose 5-phosphate, Ribo-5-P: ribose 5-phosphate, Xyl-5-P: xylulose 5-phosphate, GAP: glyceraldehyde 3-phosphate, F-6-P: fructose 6-phosphate, DHA: dihydroxyacetone, DHAP: dihydroxyacetone phosphate, Ery-4-P: erytrose 4-phosphate, S-1,7BP: sedoheptulose 1,7-bisphosphate, HCHO: acetaldehyde, GMP: guanosine monophosphate, AMP: adenosine monophosphate, IMP: inosine monophosphate, XMP: xanthosine monophosphate, OMP: orotidine monophosphate, UMP: uridine monophosphate.

**Fig. 4:**
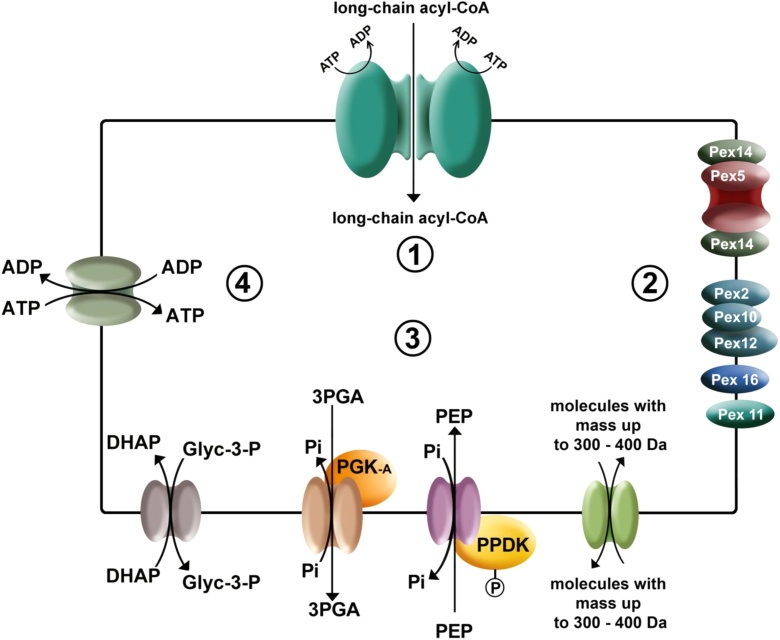
Glycosome membrane proteins detected or proposed. 1. Dimer of two half-size ABC transporters, 2. Peroxins, 3. Pore-forming proteins, 4. Proteins related to the mitochondrial carrier family (MCF).

The analysis of the glycosomal preparations showed the presence of known and novel glycosomal matrix and membrane proteins, both in exponentially growing parasites and cells grown to the stationary phase (Tables SI-SVI). Based on the data obtained, we constructed schemes for predicted glycosomal metabolic pathways of *T. cruzi* epimastigotes, as shown in Fig. 1, Fig. 2, Fig. 3, Fig. 4. Classical glycolytic enzymes (from HK to PGK) already previously reported [39,40] to be present in glycosomes of *T. cruzi* were indeed detected in the proteomes of both exponential and stationary phase cells (Fig. 1). Additionally, glucokinase, a glucose-specific phosphorylating enzyme present in *T. cruzi* but not in *T. brucei* was also detected, in agreement with its PTS1 motif (AQL) and subcellular localization previously described [41]. An interesting case is constituted by PGK, which in *T. cruzi* is encoded by three genes, denominated *PGK-A*, *PGK-B* and *PGK-C*. Our previous studies showed that all three isoforms of the enzyme are expressed in epimastigotes, with both PGK-A and PGK-C present in glycosomes, the former one being associated with the membrane, and the latter isoform located in the matrix of the organelles [42]. Meanwhile, PGK-B is cytosolic and accounts for 80% of the total cellular PGK activity [43]. Interestingly, in the current analysis, three PGKs with different predicted molecular weights (44.78, 64.87 and 100.44 kDa) were detected by the mass analysis. The sequence of the 64.87 kDa PGK (accession code Tc00.1047053511419.50) has a long extension of 85 amino acids at the N-terminus. Analysis of this N-terminal extension with TMHMM software [44] predicts two transmembrane helices (formed by residues 15 to 37 and 50 to 72), indicating that the sequence of this PGK form likely corresponds with the previously reported 56 kDa membrane-associated PGK-A isoenzyme [43,45]. The 44.78 kDa protein corresponds to the previously identified 46 kDa PGK-C. Remarkably, an unusual PGK-like protein of 100.44 kDa (Tc00.1047053504153.20) was found in the pellet obtained by treatment of the glycosomal membrane preparation with sodium carbonate. Using the TMHMM software, we could establish in this PGK the presence of three candidate transmembrane helices. These correspond to residues 745 to 767, 787 to 805 and 820 to 842 in the C-terminal region. Also intriguing was the finding of two glycosomal PGK-like proteins of 58.4 kDa with a PAS domain (Tc00.1047053506945.20 and Tc00.1047053506835.70). PAS domains are important signaling modules that can monitor changes in light, redox potential, oxygen, small ligands, and overall energy level of a cell. These domains have been identified in proteins from all three kingdoms of life: *Bacteria*, *Archaea*, and *Eukarya* [[46], [47], [48]]. This PGK-like protein with a PAS domain was previously also found in an analysis of glycosomal membrane proteins of *Leishmania tarentolae* [49].

### 3.3 Pentose-phosphate pathway

Enzymes of both the oxidative branch and the non-oxidative branch of the PPP have been detected previously in glycosomes of the three developmental forms of *T. cruzi* [50]. However, in our analysis, only transaldolase was detected in the supernatant obtained after sodium carbonate treatment of glycosomes from exponentially growing parasites. Intriguingly, we found sedoheptulose-1,7-bisphosphatase, having the PTS1 motif SKL, in both exponential and stationary phase cells. This enzyme has previously been predicted by genome analysis in *T. cruzi* and *T. brucei* [51]. Sedoheptulose-1,7-bisphosphatase is a Calvin cycle enzyme that is usually found in plastids of plants, algae, some fungi and ciliates [52]. The presence of this enzyme suggests that possibly the parasites employ this enzyme as part of a modified PPP (Fig. 3). Special attention is deserved for the presence of a hypothetical protein (Tc00.1047053511003.190) with a PTS1-like motif (SNL) that shows similarity with glucose-6-phosphate-1-epimerases. Since glucose-6-phosphate dehydrogenase is specific for β-D-glucose 6-phosphate (G6P), the presence of a glucose-6-phosphate-1-epimerase might be important for the PPP. This has been hypothesized by Gualdrón-López et al. [13] for trypanosomatids, especially for *T. brucei* which lacks a glucokinase gene. Glucokinase and hexokinases have each a preference for distinct anomers of glucose, the beta and alpha anomer, respectively. Although *T. cruzi* contains both enzymes, it cannot be ruled out that glucose-6-phosphate-1-epimerase can also participate in the regulation of the interconversion rate between the G6P anomers inside the glycosomes of *T. cruzi* [53].

### 3.4 Sugar-nucleotide synthesis

Several enzymes involved in the synthesis of sugar nucleotides were detected in the glycosome preparations. First, two isoforms of phosphomannose isomerase (Tc00.1047053503677.10 and Tc00.1047053511717.90), both enzymes having a PTS1 motif (AHM and AHI, respectively) and previously found to be essential for *T. brucei* growth [54]. The enzyme is involved in the reversible isomerization of fructose 6-phosphate to mannose 6-phosphate.

Another enzyme detected is a phosphomanno mutase-like protein (Tc00.1047053506405.10). This enzyme was previously found in glycosomes of *T. brucei* and has the pecularity of acting as a phosphoglucomutase during glycolysis [49,55]. Other enzymes of the sugar-nucleotide synthesis with PTS motifs were also found (Table SIII). Synthesis of glycoconjugates requires a constant supply of sugar nucleotides (NDP-sugars), molecules that are needed for parasite survival, invasion and/or evasion of the host immune system [56]. Indeed, galactokinase (GALK), the first enzyme involved in the salvage pathway of UDP-galactose was found associated with the glycosomal preparation. It is present in *T. cruzi* but not in *T. brucei,* as two isoenzymes of 51–52 kDa, encoded by distinct genes (*GALK-*1 and *GALK-*2) both having a PTS1 (SNL and GKL) [57].

### 3.5 Auxiliary pathways of carbon metabolism

Trypanosomatids contain enzymes for succinic fermentation, forming an auxiliary branch to the glycolytic and gluconeogenic pathways. The branch contains PEPCK, malate dehydrogenase (MDH), fumarate hydratase (FH – also known as fumarase) and a soluble NADH-dependent fumarate reductase (FRD) [3,58]. This branch was previously located in glycosomes of *T. brucei* [3]. Indeed the enzymes were also detected in our *T. cruzi* glycosomal preparation (from both exponential and stationary phase cells). Noteworthy, the glycosomal localization of one of the enzymes of the branch, FH, has been subject to discussion; this enzyme appeared to be predominantly present in the cytosol of *T. brucei* [3,37,59,60]. In addition, no authentic PTS motif was found in FH, but the presence of a cryptic PTS1 at the C-terminal extremity of *T. brucei* FH (AKLV), as well as in *L. major* FH (SKTLA) and *T. cruzi* FH (SKLL) has been suggested as a possible explanation for a dual localization in cytosol and glycosomes [61]. However, data from our current study indicate that the FH of some *T. cruzi* strains indeed possess a cryptic PTS1 (SKLL or SKLF), whereas in other strains such a cryptic PTS1 is missing (the FH sequence ending in QQLK). Interestingly, a potential PTS2 (KYVPLIPHV) was found near the N-terminus of the FH lacking the cryptic PTS1 of these latter strains (*e.g.* in the product of gene Tc00.1047053509879.40).

Another important auxiliary branch involves the enzyme PPDK that may play a role in the adenine-nucleotide homeostasis inside glycosomes, coupled to inorganic pyrophosphate (PPi) metabolism of trypanosomes. PPDK as well as an adenylate kinase [12] were detected in our glycosomal preparations. In *T. cruzi* epimastigotes a PPDK of 100 kDa can be post-translationally modified by phosphorylation and proteolytic cleavage into an inactive protein of 75 kDa. This modified form of PPDK was located peripherally at the glycosomal membrane, oriented towards the cytosol [23,62]. Meanwhile, three isoforms of adenylate kinase were detected in the proteomic analysis. One of them (Tc00.1047053509733.180) contains a PTS1-type sequence (CKL) at its C-terminus.

Interestingly, we identified two possible novel auxiliary routes for the regeneration of NAD^+^ in glycosomes. First, a D-isomer specific 2-hydroxyacid dehydrogenase (α-HADH, EC 1.1.99.6); this enzyme is a NAD-linked oxidoreductase whose catalytic properties resemble those of mouse and rat LDH-C4. This enzyme has two molecular forms (HADH-isozyme I and HADH-isozyme II). Isozyme I is responsible for the weak lactate dehydrogenase activity found in *T. cruzi* extracts [40,63,64] (Fig. 1, enzyme 16) and it might be an alternative for the reoxidation of glycosomal NADH through the reduction of the pyruvate produced by the PPDK.

The second enzyme potentially playing a role in glycosomal NAD^+^ regeneration is a PTS1-containing putative aldehyde dehydrogenase (EC 1.2.1.3) (Tc00.1047053507641.60) that could reduce and decarboxylate pyruvate to acetaldehyde (Fig. 1, enzyme 17). This enzyme was detected only in glycosomes from parasites in the exponential growth phase. Its mammalian homolog has been described as part of the α-oxidation process in peroxisomes catalyzing the oxidation of pristanal to pristanic acid (which can enter the β-oxidation pathway) [65]. Additionally, the presence of these enzymes was also reported in the proteomic analysis of purified glycosomes of *T. brucei* [22]. Interestingly, an enzyme detected in both exponential and stationary phase glycosomes is Tc00.1047053511277.60, belonging to a class of oxidoreductases that can catalyze the reversible oxidation of ethanol to acetaldehyde (thus an alcohol dehydrogenase, ADH) with the concomitant reduction of NAD(P)^+^ (Fig. 1, enzyme 18). The presence of an ADH (also known as NADP-aldehyde reductase) in *T. cruzi* has previously been described by Arauzo and Cazzulo [66]; it was reported as a cytosolic enzyme. However, in contrast to the putative aldehyde dehydrogenase, the HADH isoenzymes and ADH do not present a PTS motif, nor have they been described by Güther et al. [22] and Vertommen et al. [37] for the *T. brucei* glycosomal proteomes. However, it should be noted that also no orthologs were detectable in the *T. brucei* genome when searching TriTrypDB. Because of the lack of a typical PTS, we cannot yet entirely exclude the possibility that the two *T. cruzi* proteins represent a cytosolic or mitochondrial (a potential mitochondrial signal peptide is predicted from the HADH-isozyme I sequence) contamination of the glycosomal sample, but the presence of a possible internal glycosomal-targeting signal or import mediated by a piggy-back mechanism through their association with other glycosomal proteins remain options. These three enzymes could thus be alternatives for regeneration of NAD^+^ when PEP is not available to follow the branch PEPCK/MDH/FH/FRD. Since, however, this proposed role is only based on bioinformatic analysis and *in vitro* assays, further studies are required to determine how these enzymes are involved in the glycosomal intermediary metabolism of *T. cruzi*.

Additionally, enzymes involved in glycerol metabolism were also detected in glycosomes from trypanosomes in both phases of growth: glycerol-3-phosphate dehydrogenase, in agreement with our previous report [4] and glycerol kinase, the latter having a PTS1 motif (AKL).

### 3.6 Ether-lipid biosynthesis

The initial evidence for identification of ether-lipid biosynthesis as a glycosomal process was the finding that activity of the first two steps of this pathway is associated with the organelles from procyclic forms of *T. brucei* [67] and promastigotes of *L. mexicana* [68].

From the proteomic data obtained here on purified *T. cruzi* glycosomes we could determine the presence of alkyl-dihydroxyacetone phosphate synthase (Tc00.1047053503815.10), the second enzyme of the pathway (Fig. 2 and Tables SI and SIV). This enzyme, presenting a PTS1 motif (SHL), has also been reported in glycosomes of other kinetoplastids [22,68,69]. Nevertheless, the enzymes catalyzing the first and third step of this pathway, dihydroxyacetone phosphate acyltransferase and acyl/alkyl dihydroxyacetone phosphate reductase, also reported in glycosomes of *T. brucei* and *Leishmania* spp. [[67], [68], [69], [70]], were not detected in the *T. cruzi* proteomic analysis.

### 3.7 Lipid metabolism

β-Oxidation is a catabolic pathway that has been located in peroxisomes and mitochondria. In glycosomes, this route has only been reported in *T. cruzi* and *T. brucei* [12,71] and recently detected by proteomics in glycosomes of *T. brucei, L. donovani* and *L. tarentolae* [22,49]. According to our results, the *T. cruzi* glycosomes contain all enzymes involved in the β-oxidation, in both the exponential and stationary growth phase, and each of the sequences has a PTS1 or PTS2 (Fig. 2 and Table SI and SIV). We found two isoforms homologous to the trifunctional enzyme enoyl-CoA hydratase/enoyl-CoA isomerase/3-hydroxyacyl-CoA dehydrogenase (EC 4.2.1.17/5.3.3.8/1.1.1.35) (Tc00.1047053507547.40 and Tc00.1047053508441.70), both containing a PTS2 (RVDTILSHV), whereas the 3-ketoacyl-CoA thiolase (Tc00.1047053509463.30) presents a PTS1-like sequence (ANI).

An acyl-CoA-binding protein (ACBP) (Tc00.1047053510877.55) also detected in the *T. cruzi* glycosomal proteome shows a typical PTS1 (SKL). It was previously also found in the high-confidence proteomic analysis of *T. brucei* glycosomes [22]. ACBP binds medium- and long-chain acyl-CoA esters and has been reported to be essential in bloodstream-form *T. brucei* [72].

### 3.8 Purine and pyrimidine synthesis

While mammalian cells can synthesize purines *de novo*, protist parasites including *T. cruzi* and other trypanosomatids such as *T. brucei* and *Leishmania* spp. have to recycle them through the purine salvage pathway for the synthesis of AMP and GMP [73,74] (Fig. 3). In our proteomic analysis, three enzymes of the purine salvage pathway were detected: adenine phosphoribosyltransferase (APRT), hypoxanthine-guanine phosphoribosyltransferase (HGPRT) and inosine-5′-monophosphate dehydrogenase (IMPDH). For APRT, four isoenzymes were identified, all with a PTS1 motif (Tc00.1047053507519.150, Tc00.1047053508207.70, Tc00.1047053507519.140 and Tc00.1047053508207.74). The mass analysis detected also four isoenzymes of HGPRT (EC 2.4.2.8) (Tc00.1047053506457.40, Tc00.1047053509693.80, Tc00.1047053509693.70 and Tc00.1047053506457.30), but only two of them showed a PTS1 (AHL). For IMPDH the mass analysis indicated the presence of two isoforms (Tc00.1047053507211.40 and Tc00.1047053511301.110) which have high (98%) sequence identity and an unequivocal PTS1 (SKL). Additionally, two isoenzymes of guanosine monophosphate reductase (EC 1.7.1.7) were detected in the glycosomal fraction (Tc00.1047053506519.130 and Tc00.1047053508909.20), both with a PTS1 (SKL). Importantly, these enzymes have also been detected in the proteomic analysis of *T. brucei* glycosomes [22] (Table SI).

The nucleotide salvage pathway provides an alternative that is energetically more efficient than *de novo* synthesis for the parasites. AMP deaminase, which interconverts AMP and IMP, was also found in *T. cruzi* glycosomes and this enzyme contains a PTS1 (SRL). Similarly, this enzyme was detected in glycosome proteomic studies of other trypanosomes [22].

Unlike the situation for purines, *de novo* synthesis of pyrimidines is performed by trypanosomatids. In contrast to mammalian cells, *de novo* synthesis of UMP in glycosomes involves two enzymatic steps: by orotate phosphoribosyltransferase and orotidine-5-phosphate decarboxylase. These two activities are carried out by a bifuctional enzyme with a C-terminal SKL motif encoded by a single fused gene [9,75]. The activities were found to be associated with the glycosomes of *T. brucei* and the organellar localization was confirmed in proteomic studies [60,76]. In our analysis of *T. cruzi* glycosomes, two isoforms of the bifunctional orotate phosphoribosyltransferase/orotidine-5-phosphate decarboxylase enzyme were detected, with predicted molecular weights of 29 and 50 kDa, respectively, but only the latter one showed the classical PTS1 (SKL).

Additionally, a hypothetical protein (Tc00.1047053504147.10) detected in the mass analysis showed 98% identity with a protein putatively annotated in the TriTryp database as a phosphoribosylpyrophosphate synthetase present in other strains of *T. cruzi* (TCSYLVIO_004767 and MOQ_001239).

### 3.9 Sterol synthesis pathway

The sterol synthesis pathway, also known as the isoprenoid pathway, is an essential metabolic pathway present in eukaryotes, archaea, and some bacteria. Interestingly, peroxisomes of eukaryotes contain a set of enzymes involved in cholesterol biosynthesis that previously were considered to be cytosolic or associated with the endoplasmic reticulum. Moreover, some of them contain a conserved putative PTS1 or PTS2, supporting the notion of targeted transport into peroxisomes [77].

Our analysis detected, associated with glycosomes, a 55 kDa hypothetical protein (Tc00.1047053511903.40) that shows similarity with 3-hydroxy-3-methyl-glutaryl Coenzyme A (HMG-CoA) synthase. HMG-CoA synthase, catalyzing the first step of the mevalonate pathway, was previously located in peroxisomes [78,79]. Another key enzyme located in glycosomes is HMG-CoA reductase, which is consistent with our previous report where 80% of the activity of this enzyme was associated with glycosomes [27]. The next enzyme of this pathway, mevalonate kinase, has been shown to be almost exclusively located in glycosomes of *T. brucei* and *L. major* [80] and, in the proteomic analysis of *T. brucei*, reported with high confidence as a glycosomal protein [22]. In our analysis, this enzyme is present as two isoforms (Tc00.1047053436521.9 and Tc00.1047053509237.10), but only the latter possesses a PTS1 motif (AKI). Other enzymes of the pathway, such as isopentenyl-diphosphate Δ-isomerase (EC 5.3.3.2) (Tc00.1047053510431.10 and Tc00.1047053408799.19), squalene monooxygenase (Tc00.1047053509589.20), lanosterol 14-α-demethylase (Tc00.1047053506297.260), NAD(P)-dependent steroid dehydrogenase protein (Tc00.1047053510873.10), sterol 24-C-methyltransferase (Tc00.1047053505683.10), C-8 sterol isomerase (Tc00.1047053510329.90) and sterol C-24 reductase (EC 1.3.1.71) (Tc00.1047053507709.90) were also detected (Table SI and SIV). Of these enzymes, only isopentenyl-diphosphate Δ-isomerase and C-8 sterol isomerase were detected in proteomic analyses of glycosomes purified from procyclic-form *T. brucei* [22] and only isopentenyl-diphosphate Δ-isomerase, squalene monooxygenase and C-8 sterol isomerase present PTS1 or PTS2 motifs. Interestingly, HMG-CoA reductase, squalene synthase [27,81], sterol 24-C-methyltransferase and mevalonate kinase were already previously reported as enzymes of the mevalonate pathway present in glycosomes of trypanosomes [60,82]. It should be noted that in analysis of peroxisomes from mammals, phosphomevalonate kinase [83], isopentenyl diphosphate isomerase [84], acetoacetyl-CoA thiolase [85], HMG-CoA synthase [79], HMG-CoA reductase [86], mevalonate kinase [87], mevalonate diphosphate decarboxylase [83], and farnesyl diphosphate synthase [88] were all found to possess a PTS motif and their peroxisomal localization has been determined experimentally. Interestingly, the acetoacetyl-CoA thiolase (EC 2.3.1.9) from mammals that condenses two molecules of acetyl-CoA to give acetoacetyl-CoA, contains both a mitochondrial signal peptide at the amino terminus and a PTS1 at the carboxy terminus [79]. In our mass analysis, we detected the presence of several of the enzymes involved in this anabolic pathway (Fig. 2); some of them have a clear PTS1 motif, but others showed neither a PTS1 or PTS2. The presence of these latter enzymes in glycosomes may be attributed to the existence of non-consensus targeting sequences or piggy-back transport.

### 3.10 Detoxification of oxygen radicals

Iron-dependent superoxide dismutase (SOD) is an enzyme involved in the dismutation of the highly reactive, toxic superoxide radical with the formation of hydrogen peroxide (H_2_O_2_). It has been detected in various kinetoplastids [89]. Four distinct SOD activities (SOD I, II, III, and IV) have been characterized in the epimastigote form of *T. cruzi* and selective membrane permeabilization with digitonin showed that these SODs are primarily cytosolic, with small amounts associated to glycosomes (SOD I and II of 20 and 60 kDa, respectively) and mitochondrion (SOD III of 50 kDa) [90]. In our proteomic analysis two Fe-SODs of 23.5 kDa were identified (Tc00.1047053508445.20 and Tc00.1047053511715.10) (Table SI). Their presence and location are consistent with the glycosomal proteome of procyclic *T. brucei* [22,60], and previous reports about the characterization of these isoenzymes in *T. brucei* [91,92].

Additionally, two isoenzymes of tryparedoxin peroxidase were detected in the glycosomal fraction (Tc00.1047053487507.10 and Tc00.1047053509499.14). These enzymes participate in decomposing H_2_O_2_ using electrons donated either directly from trypanothione, or *via* the redox intermediate tryparedoxin. Is important to mention the detection of a glutathione peroxidase-like protein (Tc00.1047053503899.130) with a PTS2 motif in the glycosomal mass analysis that could contribute to this process. Tryparedoxin (Tc00.1047053509997.30), having a predicted PTS2, was also detected in the analysis. Additionally, this protein was reported in the glycosomal proteome of *T. brucei* (Tb927.3.3760) [22]. The flavoprotein trypanothione reductase is a key enzyme in the antioxidant metabolism of trypanosomes and represents a potential drug target. This enzyme from various kinetoplastid parasites has been characterized in detail [[93], [94], [95]]. In our analysis two isoenzymes of 42 and 54 kDa were detected (Tc00.1047053504507.5 and Tc00.1047053503555.30, respectively) with the latter presenting a PTS1 (ASL). The presence of this enzyme in glycosomes is corroborated by the glycosomal proteome of *T. brucei* [22].

### 3.11 Gluconeogenesis

Two fructose-1,6-bisphosphatase (FBPase) isoforms were detected in the proteomic analysis of *T. cruzi* glycosomes (Tc00.1047053506649.70 and Tc00.1047053508351.10); they show 98% sequence identity and have both a classical PTS1 (SKL). This is a hallmark enzyme of gluconeogenesis whose presence was previously reported in an extract of epimastigotes of *T. cruzi* and in the proteomic analyses of glycosomes of *T. brucei* and *L. donovani* [22,96]. Interestingly, FBPase and PFK can be expressed simultaneously in glycosomes of *T. cruzi* and other kinetoplastids, potentially so creating a futile cycle causing loss of ATP. However, activating the FBPase may also function to partially re-direct the glucose 6-phosphate from glycolysis to the PPP, as has been shown in *Toxoplasma* and hypothesized for *Leishmania* [97]. Nonetheless, studies with *T. brucei* are suggestive of a reciprocal regulation in the activities of these enzymes possibly by post-translational modification.

### 3.12 Transporters/integral membrane proteins

In peroxisomal membranes various types of transporters have been described: peroxins, half-size ABC transporters, pore-forming proteins and proteins of the mitochondrial carrier family (MCF). More recently peroxins and ABC transporters have also been identified in glycosomal membranes, and the existence of pore-forming proteins was also established, however the identity of these latter proteins remains to be established [6].

Peroxins (also known with the acronym PEX) are a group of proteins involved peroxisome biogenesis, including the process of matrix-protein import [98]. Most of them are integral or peripheral membrane proteins, whereas some are soluble cytosolic proteins, either permanently or transiently interacting with the membrane. For trypanosomatids, peroxins have been studied in detail in *T. brucei* [20] and several of them were also identified in its glycosomal proteome [22]. In our proteomic analysis of *T. cruzi* purified glycosomes, we also detected several PEX proteins (Table SI). First, peroxins implicated in matrix protein import such as PEX2 (Tc00.1047053508479.230), PEX10 (Tc00.1047053508479.190), PEX12 (Tc00.1047053503809.20) were detected in the pellet fraction of glycosomes treated by osmotic shock and with Na_2_CO_3_. PEX14 (Tc00.1047053511145.40) was equally detected in the pellet fraction of glycosomes treated by osmotic shock. A search for proteins that align with the sequence of Tc00.1047053503811.40 detected in the pellet of the Na_2_CO_3_ treated glycosomes, showed 75% identity with the PTS2 receptor PEX7 of *T. brucei* (Tb03.28C22.1010) [99].

Other integral membrane proteins that were detected are a protein annotated as (Tc00.1047053510043.40) that presents 33% identity with *T. brucei* PEX16 [100], *i.e.* a peroxin involved in insertion of peroxisomal membrane proteins (PMPs), as well as homologs of *T. brucei* PEX11 (Tc00.1047053504005.40) and GIM5A (Tc00.1047053507009.10) that is related to PEX11 [101]. PEX11 is known to be involved in the morphology establishment and proliferation of peroxisomes, but has also been identified, in yeast, as a pore-forming protein [102].

The second group of known transporters in the glycosomal membrane of *T. brucei* comprises three homologs of peroxisomal half-size ABC transporters designated GAT1-3 (for glycosomal ABC transporters 1 to 3) [103]. Only for GAT1 a function has been shown, *i.e.* the transport of acyl-CoAs from the cytosol into the glycosomal lumen [104]. The function of GAT2 and GAT3 has not yet been established. Interestingly, in the membrane fraction of *T. cruzi* glycosomes treated with Na_2_CO_3_, various ABC transporters were detected (Tables SI and SVI). Three ABC transporters show a considerably high identity with GAT1, 2 and 3 of *T. brucei*. The protein (Tc00.1047053508927.20), annotated as a hypothetical glycosomal transporter in the database, shows 49% identity with TbGAT1 (Tb927.4.4050), whereas the other ABC transporters detected (Tc00.1047053510431.150 and Tc00.1047053506925.530) show 52% and 54% identity with GAT2 (Tb11.02.0630) and GAT3 (Tb11.03.0030) of *T. brucei*, respectively. The three proteins were also reported with a high confidence as glycosomal proteins in the *T. brucei* proteomics analysis [22].

As a third group of molecules involved in the transport of metabolites through the peroxisomal and glycosomal membrane are pore-forming proteins. Such pores have been well characterized for peroxisomes, notably from mammalian cells and, besides PEX11, one other pore-forming protein has been identified [105]. The sizes of the peroxisomal pores have been determined electrophysiologically and will allow permeation of molecules with a Mr up to about 300–400 Da. Furthermore, three main channel-forming activities were detected in membranes of the glycosomal fraction from bloodstream-form *T. brucei* permitting currents with amplitudes 70–80 pA, 20–25 pA, and 8–11 pA, respectively [5]. In analogy to the situation in peroxisomes, it has been proposed that such pores would allow the glycosome entry or exit of metabolites of low molecular masses such as inorganic ions including phosphate, PPi, glucose, G6P, oxaloacetate, malate, succinate, PEP, 1,3-bisphosphoglycerate, 3PGA, DHAP, Glyc3P, *etc*., but not larger molecules such as ATP, ADP, NAD(P)^+^, NADH(P), CoA, and fatty acids or acyl-CoAs [5,105].

In our glycosome analysis, two proteins (Tc00.1047053509197.20 and Tc00.1047053508699.130) were identified as cation transporters (Table SI). However, for the first one, no additional report is available as yet to confirm it being localized in glycosomes, whereas the second protein was previously identified as part of the contractile vacuole complex of *T. cruzi* [106] and may thus have been a contamination in the glycosomal fraction.

In addition, a transporter protein involved in maintaining the redox balance within glycosomes through a Glyc3P/DHAP shuttle-based mechanism has not yet been described but its existence is presumed [1,107]. Translocation of amino acids across the glycosomal membrane is also necessary; it is likely that amino acids could diffuse through the glycosomal pores. Nonetheless, the plasma-membrane arginine transporter AAP3 has, under certain culturing conditions, also been detected in the membrane of *Leishmania* glycosomes [108,109].

As shown in Tables S1 and SVI, two proteins (Tc00.1047053508551.39 and Tc00.1047053506355.10) were found in the pellet fraction obtained after carbonate treatment of glycosomal membranes. Their sequences are identical to that of TcHT, previously identified as the plasma-membrane glucose transporter by Silber et al., 2009 [110]. Intriguingly, based on their immunolocalization studies, these authors reported the association of this transporter also with glycosomes and reservosomes. However, the functionality of TcHT as a hexose transporter in the glycosomal membrane seems doubtful, not only because so far no evidence has been found for functional transporters for small (<400 Da) solutes in the membrane of peroxisome-related organelles of any organism – the current notion is that translocation occurs *via* channels – but also because membrane insertion of proteins of plasma-membrane and peroxisomes/glycosomes is known to occur by distinct mechanisms, involving specific topogenic signals and proteins. Even when similar proteins would be routed to these different destinations after insertion into the endoplasmic reticulum membrane, they would have opposite topologies in the plasma membrane and glycosomal membrane. Additionally, it would be difficult to imagine how, in view of the different extra- and intracellular glucose concentrations, transport of this substrate across the different membranes would be mediated by carriers with identical kinetic properties. A possible explanation for the finding of TcHT also in intracellular membranes is a non-specific association of excess transporter molecules with hydrophobic environments, either directly with organellar membranes or after association with the specific domains of the endoplasmic reticulum destined to be routed to proliferating glycosomes. This same explanation may be invoked for the *Leishmania* arginine plasma-membrane transporter AAP3 that, under certain conditions is also found associated with glycosomes as mentioned above.

Previously, a protein profile of glycosomal membranes from *T. cruzi* epimastigotes revealed a most abundant protein of 75 kDa which was later identified as PPDK [23,62]. This protein was initially detected in the glycosomal membrane pellet obtained after treatment with Na_2_CO_3_, but also in the soluble phase confirming its dual location in both the membrane and the glycosomal matrix [23]. Interestingly, the PPDK associated to the glycosomal membrane (75 kDa) appeared to be post-translationally modified by phosphorylation and proteolytic cleavage [62]. Moreover, studies of *T. cruzi* epimastigotes permeabilized with digitonin and incubated with anti-PPDK showed significant inhibition of glucose consumption when PEP was included in the assay. This result may indicate the participation of the 75 kDa form of PPDK in the entry of PEP into glycosomes (our unpublished results).

Several studies have indicated that *T. brucei* HK has a strong tendency to remain associated with the glycosomal membrane [20]. Some authors even suggested that the entry of glucose into the glycosomes and the association of HK to the glycosomal membrane might be functionally linked. Interestingly, oligomycin inhibits the linkage *via* a mechanism that appeared not to be correlated to the energy charge of the cell [111]. In addition, our experience in the purification of *T. cruzi* HK showed that when a glycosome-enriched fraction was treated with 80% ammonium sulfate (saturated concentration), the enzyme’s activity was recovered as a flocculate floating on the solution (no protein pellet was ever observed at this stage) [112]. This latter observation would support the notion that HK of *T. cruzi* might be associated with insoluble regions of the membrane, possibly in association with a pore as has been proposed [20]. Further support for the hypothesis is the detection, in our mass analysis, of HK in both the soluble fractions and the pellets obtained after treatment with Na_2_CO_3_ and osmotic shock.

Other evidence that might suggest a close association between some enzymes and glycosomal transporters or pores results from studies performed on isolated glycosomes. In these studies the glycosomal latency values of enzymes showed some inconsistencies. For example, assays performed on glycosomes from *T. cruzi* and *T. brucei* showed high latency for HK (between 77–80%), whereas in these same preparations PGK showed only very low or no latency (3–50%) [2,113], our unpublished results]. A reinterpretation of these data could be that the glycosomal PGK is tightly associated with the membrane, for example forming a complex with a pore to facilitate specifically the translocation of 3PGA between the glycosomal matrix and the cytosol.

A third group of peroxisomal membrane proteins is formed by some MCF representatives [[114], [115], [116]]. Three different proteins of this family have been reported in the glycosomal proteome of *T. brucei* [22,61]. These are homologs of transporters of phosphate, dicarboxylates and ATP/ADP exchange. However, the unambiguous localization of MCF transporters in glycosomes and the assignment of substrate specificity for such transporters in general and trypanosomes in particular is not always evident as shown in functional studies [117]. Nonetheless, a MCF transporter with broad specificity for different, bulky compounds such as ATP, ADP, AMP, CoA and NAD^+^ has been identified in human, yeast and plant peroxisomes [116,[118], [119], [120], [121], [122]]. In our mass analysis we detected homologs of a mitochondrial phosphate transporter (Tc00.1047053509551.30) as well as an ADP/ATP mitochondrial carrier protein (Tc00.1047053511249.10) which have high identity (close to 85%) with a protein annotated as mitochondrial carrier protein 11 (Tb09.211.1750) and ADP/ATP mitochondrial translocase (Tb927.7.3940), respectively, in the *T. brucei* genome database. However, the functions of these proteins in *T. brucei* have not yet been proved. Interestingly, both proteins were reported as high-confidence glycosomal proteins in the proteomic analysis of *T. brucei* [22]. The ADP/ATP mitochondrial carrier homolog of *T. cruzi* presents a putative mitochondrial carrier sequence signature as well as the non-canonical mitochondrial ATP/ADP motif sequence RRRMMM. It is attractive to speculate that this protein, if (also) present in the glycosomal membrane, could provide additional ATP necessary for intra-glycosomal biosynthetic processes when the PGK and PEPCK would not be able to provide it. In contrast to other antiport systems, the ADP/ATP translocase is an electrogenic antiporter exchanging ATP that has four negative charges against ADP with only three, resulting in the net translocation of a negative charge. This translocator has been characterized in the inner mitochondrial membrane where the electrochemical proton gradient is the driving force for the exchange of ATP and ADP. In the case of glycosomes such translocator would be expected to act in most instances to import ATP – thus in the opposite direction of its normal mitochondrial function (although as shown for the *T. brucei* mitochondrion, it can be reversed under certain conditions [123] and the output of inorganic phosphate would be essential.

Another protein found is a homolog of a tricarboxylate carrier (Tc00.1047053511583.40) of 36.2 kDa. A homolog has also been detected by proteomic analysis of the glycosomal membrane of *T. brucei* (Tb927.9.4310) and *L. tarentolae* (LtaP01.0560) [49]. This putative tricarboxylate carrier of *T. cruzi* showed 69% and 56% identity with the corresponding protein of *T. brucei* and *L. tarentolae*, respectively. Furthermore, we detected a protein (Tc00.1047053506773.90) of 51 kDa with 62% identity with a putative amino-acid permease/transporter of *T. brucei* (Tb11.02.4520) and two other candidate amino-acid transporters (Tc00.1047053506153.10 and Tc00.1047053507585.10), each possessing 11 predicted transmembrane helices.

It should be realized that for many of the glycosomal membrane proteins, whether integral or peripheral, identified in the proteome analyses of both *T. cruzi* and other trypanosomatids, further studies will be required to prove unambiguously that they are authentic glycosomal (or shared between glycososomal and other membranes such as that of the mitochondrion) and not contaminants in the glycosomal fractions analyzed. Moreover, functional studies to prove the identities of the membrane proteins have only been performed for the peroxins (mostly for *T. brucei*, to a lesser extent for *Leishmania* spp.) and one of the *T. brucei* ABC transporters (GAT1). For all other proteins discussed above, the possible functional identity is predicted from homology with molecules in non-trypanosomatid organisms. However, it is important to realize that glycosomes have a very different metabolic repertoire than peroxisomes of other organisms, so they need different metabolite transporters. Trypanosomatids may have achieved this by recruiting appropriate transporters from the available repertoire in other membranes such as the mitochondrial inner membrane and/or by changing the substrate specificity of transporters inherited from the peroxisomes that evolved into glycosomes [5]. Functions assigned solely based on sequence comparison should therefore be considered as tentative and require confirmation by future functional studies.

### 3.13 Hypothetical proteins detected in association with glycosomes

A variety of other proteins bearing a classical PTS1 but with unknown function were detected in our proteomic analysis (Table SII). Several of these proteins present high identity with hypothetical proteins identified with high confidence as glycosomal proteins in the proteomic analysis of *T. brucei* [22]. Moreover, using a transmembrane domains predictor TMHMM software, we could establish that several of these hypothetical proteins present potential transmembrane alpha-helical segments. These proteins have a variable number of alpha-helices, ranging between one and 17 (Table SII). This characteristic suggests that these proteins, passing several times through the membrane, might fulfill the function of channels or transporters of important ions or molecules involved in the metabolism occurring within glycosomes.

An interesting finding is the presence of a protein with 58.9% identity to the *T. brucei* serine/threonine phosphatase PIP39 (Table SVI; Tc00.1047053509353.40). Moreover, it has an identical PTS1 as TbPIP39 (SRL). TbPIP39 is part of a protein phosphatase cascade that regulates differentiation between the parasite’s developmental forms [124]. Prior to the trigger for differentiation, TbPIP39 is kept inactivated in a cytosolic assembly with the tyrosine phosphatase TbPTP1, that dephosphorylates it. Upon the differentiation trigger – the uptake of citrate from the blood into the trypanosome – the PTP1/PIP39 complex is disrupted and PIP39 becomes phosphorylated and activated and is translocated to the glycosomes. Future studies may reveal if PIP39 and glycosomes are also involved in life-cycle differentiation of *T. cruzi*.

## 4 Concluding remarks

In this manuscript we have presented insight into the different biochemical processes that are carried out in the glycosomes of *T. cruzi* epimastigotes through a proteomic analysis of a purified fraction containing this organelle from cells grown to an exponential and stationary phase. The intraglycosomal enzymatic equipment and the proteins present in the glycosomal membrane reveal low variations between these two growth phases of *T. cruzi* epimastigotes indicating that the protein composition of this organelle is – at least in a qualitative sense – largely independent of the carbon source. In addition to known glycosomal routes, we have found new glycosomal constituents which were identified by electrospray ionization mass spectrometry (ESI) in an Orbitrap Elite MS (Thermo Scientific).

These results provide information on possible new metabolic pathways in the organelles, as well as the identification of proteins possibly present in the glycosomal membrane that could fill the void of solute translocation mechanisms necessary for metabolism within the glycosomes. Several enzymes were found that had not yet been reported as glycosomal but have motifs such as PTS1 and PTS2, *e.g.* aldehyde dehydrogenase, PAS-domain containing PGK, nucleoside diphosphate kinase, arginine kinase, ribokinase, L-ribulokinase, sedoheptulose-1,7-bisphosphatase, dihydroxyacetone kinase 1-like protein, and phosphoribosylpyrophosphate synthetase. To corroborate the glycosomal localization of the new components identified in this study, it is necessary to carry out complementary experiments such as immunolocalization as well as others that allow to clarify the presence of the respective enzymes.
