# Supplementary material for: Proteomic analysis of glycosomes from Trypanosoma cruzi epimastigotes
Source: Mol Biochem Parasitol. 2019 Apr;229:62–74. doi: 10.1016/j.molbiopara.2019.02.008 (PMC7082770; doi:10.1016/j.molbiopara.2019.02.008)
Supplement: Supplementary file 1 [file mmc1.docx]

**SUPPLEMENTARY INFORMATION**

**PROTEOMIC ANALYSIS OF GLYCOSOMES FROM *Trypanosoma cruzi* EPIMASTIGOTES**

**Héctor Acosta^a^, Richard Burchmore^b^, Christina Naula^b^, Melisa Gualdrón-López^c^,** **Ender Quintero-Troconis^a^, Ana J. Cáceres^a^, Paul A. M. Michels^d^**, **Juan Luis Concepción^a^, Wilfredo Quiñones^a*^**

*^a^Laboratorio de Enzimología de Parásitos, Facultad de Ciencias, Universidad de Los Andes, Mérida 5101, Venezuela.*

*^b^Institute of Infection, Immunity and Inflammation, College of Medical, Veterinary and Life Sciences, University of Glasgow, Glasgow, G12 8QQ, UK.*

^c^*Present address:Instituto de Salud Global de Barcelona (ISGlobal), Rosselló, 132, 4.08036, Barcelona, Spain.*

*^d^Centre for Immunity, Infection and Evolution and Centre for Translational and Chemical Biology, The University of Edinburgh, Edinburgh EH9 3FL, UK.*

^*^**Corresponding author** Tel: +58 274 2401302; fax: +58 274 2401390; e-mail address: wilqui@ula.ve (W. Quiñones).

**
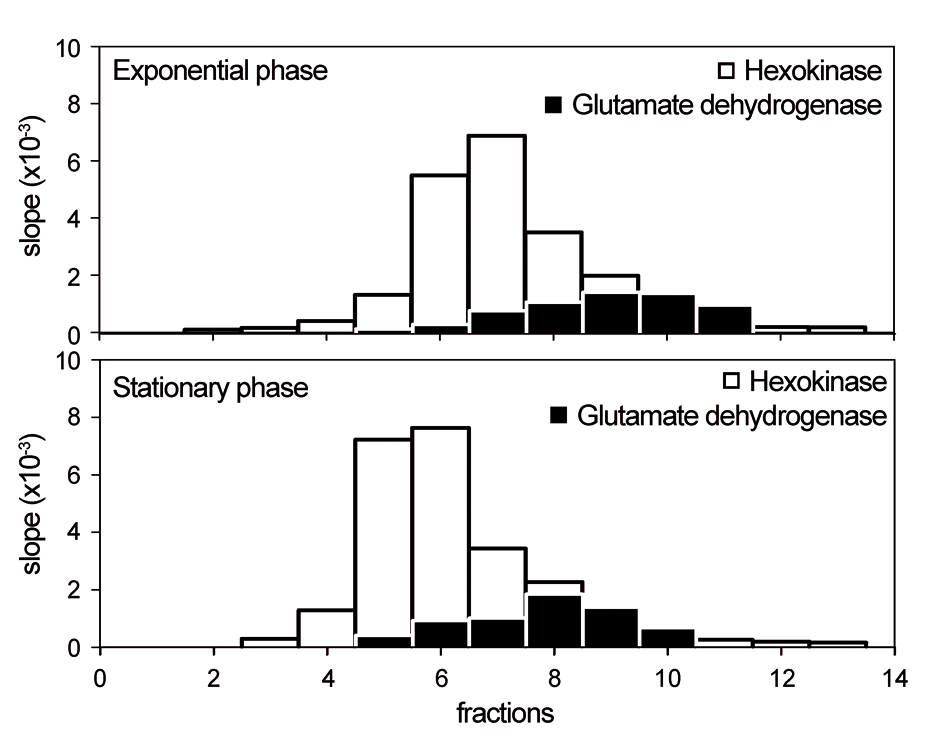
Figure S1.**

**Purification of glycosomes by isopycnic centrifugation.** 1.5 ml of a small-granular fraction (SG), obtained by differential centrifugation, was applied on top of a linear 0.25-2.5 M sucrose density gradient (1.05-1.30 g.cm^-3^) in a 35 ml tube and centrifuged at 170,000 xg for 120 min. The content of the tube was collected in 18 fractions of 1.9 ml. The activities of hexokinase as glycosomal marker enzyme (□) and glutamate dehydrogenase as mitochondrial marker enzyme (■) were measured for glycosomes purified from cells taken during the exponential and stationary growth phase in the presence of 0.1% Triton X-100 and 150 mM NaCl and expressed as slope [absorbance unit (340 nm).s^-1^].

**Table SI. Complete list of proteins identified in the proteomics analysis of *Trypanosoma cruzi* glycosomes treated with osmotic shock or Na_2_CO_3_.**

| **Accession Number** | | **Description** | | | | | | | | | | | | | | | | | | | | | **PTS** | **Osm Shock** | | | | **Na_2_CO_3_** | | | | | **Reported in glycosomes** | | | | | | | | | | | | | | |
| --- | --- | --- | --- | --- | --- | --- | --- | --- | --- | --- | --- | --- | --- | --- | --- | --- | --- | --- | --- | --- | --- | --- | --- | --- | --- | --- | --- | --- | --- | --- | --- | --- | --- | --- | --- | --- | --- | --- | --- | --- | --- | --- | --- | --- | --- | --- | --- |
|  |  |  |  |  |  |  |  |  |  |  |  |  |  |  |  |  |  |  |  |  |  |  |  | **sup** | | | **pellet** | **sup** | | **pellet** | | | **Güther*** | | | | | | | **Vertommen**** | | | | | **References** | | |
| **Glycolytic metabolism** | | | | | | | | | | | | | | | | | | | | | | |  |  | | |  |  | |  | | |  | | | |  | | | | | |  | | | | |
| **Tc00.1047053510121.20** | | hexokinase | | | | | | | | | | | | | | | | | | | | | 2 | + | | | + | + | | - | | | + | | | | + | | | | | | 1,3,4 | | | | |
| **Tc00.1047053510187.100** | | glucokinase 1 | | | | | | | | | | | | | | | | | | | | | 1 | + | | | + | + | | - | | | - | | | | - | | | | | | 2 | | | | |
| **Tc00.1047053510889.221** | | glucose-6-phosphate isomerase | | | | | | | | | | | | | | | | | | | | | 1 | + | | | + | + | | - | | | + | | | | + | | | | | | 1,3,4,5 | | | | |
| **Tc00.1047053506529.508** | | glucose-6-phosphate isomerase | | | | | | | | | | | | | | | | | | | | | 1 | + | | | + | + | | - | | | + | | | | + | | | | | | 1,3,4, 5 | | | | |
| **Tc00.1047053508153.340** | | 6-phospho-1-fructokinase | | | | | | | | | | | | | | | | | | | | | 1 | + | | | + | + | | + | | | + | | | | + | | | | | | 1, 3,4 | | | | |
| **Tc00.1047053510301.20** | | fructose-bisphosphate aldolase | | | | | | | | | | | | | | | | | | | | | 2 | - | | | - | + | | - | | | + | | | | + | | | | | | 1, 3 | | | | |
| **Tc00.1047053504163.40** | | fructose-bisphosphate aldolase | | | | | | | | | | | | | | | | | | | | | 2 | - | | | + | + | | - | | | + | | | | + | | | | | | 1,3 | | | | |
| **Tc00.1047053508647.200** | | triosephosphate isomerase | | | | | | | | | | | | | | | | | | | | | - | + | | | + | + | | - | | | + | | | | - | | | | | | 1,3 | | | | |
| **Tc00.1047053506943.50** | | glyceraldehyde 3-phosphate dehydrogenase | | | | | | | | | | | | | | | | | | | | | 1 | + | | | + | + | | - | | | + | | | | + | | | | | | 1,3 | | | | |
| **Tc00.1047053511419.40** | | phosphoglycerate kinase | | | | | | | | | | | | | | | | | | | | | 2 | - | | | - | + | | - | | | + | | | | + | | | | | | 1, 6 | | | | |
| **Tc00.1047053511419.50** | | phosphoglycerate kinase | | | | | | | | | | | | | | | | | | | | | - | - | | | + | - | | *-* | | | + | | | | + | | | | | | 1, 6,7 | | | | |
| **Tc00.1047053504153.20** | | phosphoglycerate kinase | | | | | | | | | | | | | | | | | | | | | - | - | | | + | - | | *+* | | | + | | | | + | | | | | | 1, 6 | | | | |
| **Tc00.1047053506125.30** | | phosphoglycerate kinase | | | | | | | | | | | | | | | | | | | | | - | - | | | + | - | | *+* | | | + | | | | + | | | | | | 1, 6 | | | | |
| **Tc00.1047053506835.70** | | PAS-domain containing phosphoglycerate kinase | | | | | | | | | | | | | | | | | | | | | 1 | + | | | + | + | | *-* | | | - | | | | - | | | | | | - | | | | |
| **Tc00.1047053506945.20** | | PAS-domain containing phosphoglycerate kinase | | | | | | | | | | | | | | | | | | | | | 1 | + | | | - | - | | *+* | | | - | | | | - | | | | | | - | | | | |
| **Tc00.1047053511277.60** | | alcohol dehydrogenase | | | | | | | | | | | | | | | | | | | | | - | + | | | + | + | | *-* | | | - | | | | - | | | | | |  | | | | |
| **Tc00.1047053506263.20** | | D-isomer specific 2-hydroxyacid dehydrogenase-protein | | | | | | | | | | | | | | | | | | | | | - | - | | | - | + | | - | | | - | | | | - | | | | | | 8 | | | | |
| **Tc00.1047053510099.120** | | D-isomer specific 2-hydroxyacid dehydrogenase-protein | | | | | | | | | | | | | | | | | | | | | - | - | | | + | - | | - | | | - | | | | - | | | | | | 8 | | | | |
| **Tc00.1047053506263.10** | | D-isomer specific 2-hydroxyacid dehydrogenase-protein | | | | | | | | | | | | | | | | | | | | | - | - | | | - | *+* | | *-* | | | - | | | | - | | | | | | 8 | | | | |
| **Tc00.1047053506263.30** | | D-isomer specific 2-hydroxyacid dehydrogenase-protein | | | | | | | | | | | | | | | | | | | | | - | - | | | + | - | | *-* | | | *-* | | | | - | | | | | | 8 | | | | |
| **Tc00.1047053507641.60** | | aldehyde dehydrogenase | | | | | | | | | | | | | | | | | | | | | 1 | + | | | - | - | | *-* | | | + | | | | - | | | | | | 9 | | | | |
| **Tc00.1047053509065.9** | | aldehyde dehydrogenase (fragment) | | | | | | | | | | | | | | | | | | | | | - | - | | | + | - | | + | | | + | | | | *-* | | | | | | 9 | | | | |
|  | | | | | | | | | | | | | | | | | | | | | | | | | | | | | | | | | | | | | | | | | | | | | | | |
| **Gluconeogenesis** | |  | | | | | | | | | | | | | | | | | | |  | | |  | | |  |  | |  | | |  | | | |  | | | | | |  | | | | |
| **Tc00.1047053506649.70** | | fructose-1,6-bisphosphatase | | | | | | | | | | | | | | | | | | | | | 1 | - | | | + | + | | - | | | + | | | | - | | | | | | 27 | | | | |
| **Tc00.1047053508351.10** | | fructose-1,6-bisphosphatase | | | | | | | | | | | | | | | | | | | | | 1 | + | | | + | - | | + | | | + | | | | - | | | | | | 27 | | | | |
|  | | | | | | | | | | | | | | | | | | | | | | | | | | | | | | | | | | | | | | | | | | | | | | | |
| **Galactose metabolism** | | |  |  | | | | | | | | | |  | | | | | | | | |  |  | | |  |  | | | | |  | | | | | | | | | |  | | | | |
| **Tc00.1047053510667.120** | | galactokinase | | | | | | | | | | | | | | | | | | | | | 1 | - | | | + | - | | + | | | - | | | | - | | | | | | 10 | | | | |
| **Tc00.1047053507009.40** | | galactokinase | | | | | | | | | | | | | | | | | | | | | 1 | + | | | - | + | | - | | | - | | | | - | | | | | | 10 | | | | |
| **Tc00.1047053508465.90** | | galactokinase | | | | | | | | | | | | | | | | | | | | | 1 | - | | | - | + | | - | | | - | | | | - | | | | | | 10 | | | | |
| **Tc00.1047053507001.110** | | galactokinase-like protein | | | | | | | | | | | | | | | | | | | | | 1 | + | | | + | + | | - | | | - | | | | - | | | | | | 10 | | | | |
|  | | | | | | | | | | | | | | | | | | | | | | | | | | | | | | | | | | | | | | | | | | | | | | | |
| **Sugar-nucleotide synthesis** | | | | | | | | | | |  | |  | | | | |  | | | | |  |  |  | | | | |  | | | | |  | | | | | | | | | | | | |
| **Tc00.1047053506405.10** | | phosphomannomutase-like protein | | | | | | | | | | | | | | | | | | | | | 1 | - | | | + | + | | - | | | - | | | | + | | | | | | 11 | | | | |
| **Tc00.1047053511531.50** | | glucosamine-6-phosphate isomerase | | | | | | | | | | | | | | | | | | | | | 1 | + | | | + | - | | + | | | - | | | | + | | | | | | - | | | | |
| **Tc00.1047053511025.50** | | glucosamine-6-phosphate isomerase | | | | | | | | | | | | | | | | | | | | | 1 | - | | | + | + | | - | | | - | | | | + | | | | | | - | | | | |
| **Tc00.1047053511717.90** | | phosphomannose isomerase | | | | | | | | | | | | | | | | | | | | | 1 | + | | | - | + | | - | | | + | | | | - | | | | | | 12 | | | | |
| **Tc00.1047053503677.10** | | phosphomannose isomerase | | | | | | | | | | | | | | | | | | | | | 1 | + | | | + | + | | - | | | + | | | | - | | | | | | 12 | | | | |
| **Tc00.1047053506341.10** | | N-acetylglucosamine-6-phosphate deacetylase-like protein | | | | | | | | | | | | | | | | | | | | | 1 | - | | | - | - | | + | | | - | | | | - | | | | | | - | | | | |
| **Tc00.1047053506507.10** | | N-acetylglucosamine-6-phosphate deacetylase-like protein | | | | | | | | | | | | | | | | | | | | | 1 | + | | | + | + | | - | | | - | | | | - | | | | | | - | | | | |
| **Tc00.1047053510259.18** | | GlcNAc-PI synthesis protein (GPI3) | | | | | | | | | | | | | | | | | | | | | 2 | - | | | - | - | | + | | | - | | | | - | | | | | | - | | | | |
| **Tc00.1047053504557.10** | | UDP-Gal or UDP-GlcNAc-dependent glycosyltransferase, putative | | | | | | | | | | | | | | | | | | | | | 2 | + | | | + | - | | + | | | - | | | | - | | | | | | - | | | | |
| **Tc00.1047053505163.80** | | oligosaccharyl transferase subunit, putative | | | | | | | | | | | | | | | | | | | | | 2 | - | | | - | - | | + | | | - | | | | - | | | | | | 11 | | | | |
|  | | | | | | | | | | | | | | | | | | | | | | | | | | | | | | | | | | | | | | | | | | | | | | | |
| **Dicarboxylate metabolism** | | | | | | | | | | | | | |  |  | | | | | | | |  |  | | |  |  |  | | | | |  | | | | | | | | | |  |  |  |  |
| **Tc00.1047053505807.180** | | 2-hydroxy-3-oxopropionate reductase | | | | | | | | | | | | | | | | | | | | | - | + | | | - | + | | - | | | - | | | | *-* | | | | | | - | | | | |
|  | | | | | | | | | | | | | | | | | | | | | | | | | | | | | | | | | | | | | | | | | | | | | | | |
| **Auxiliary enzymes to glycolysis/gluconeogenesis** | | | | | | | | | | | | | | | | | | | | | | | | | | | | | | | | | | | | | | |  |  |  |  |  |  |  |  |  |
| **Tc00.1047053508441.20** | | phosphoenolpyruvate carboxykinase | | | | | | | | | | | | | | | | | | | | 1 | | + | | | + | + | | - | | | + | | | | *-* | | | | | | 4, 13, 14 | | | | |
| **Tc00.1047053511293.69** | | glycosomal malate dehydrogenase | | | | | | | | | | | | | | | | | | | | 1 | | + | | | + | + | | - | | | + | | | | - | | | | | | 15 | | | | |
| **Tc00.1047053509879.40** | | fumarate hydratase | | | | | | | | | | | | | | | | | | | | 2 | | + | | | - | - | | - | | | - | | | | - | | | | | | 16 | | | | |
| **Tc00.1047053510215.10** | | NADH-dependent fumarate reductase | | | | | | | | | | | | | | | | | | | | 1 | | + | | | + | + | | - | | | + | | | | - | | | | | | 16 | | | | |
| **Tc00.1047053503849.80** | | NADH-dependent fumarate reductase | | | | | | | | | | | | | | | | | | | | 1 | | + | | | + | - | | + | | | + | | | | - | | | | | | 16 | | | | |
| **Tc00.1047053503849.60** | | NADH-dependent fumarate reductase | | | | | | | | | | | | | | | | | | | | 2 | | - | | | + | + | | - | | | + | | | | - | | | | | | 16 | | | | |
| **Tc00.1047053503467.9** | | NADH-dependent fumarate reductase | | | | | | | | | | | | | | | | | | | | - | | + | | | + | - | | + | | | + | | | | - | | | | | | 16 | | | | |
| **Tc00.1047053508535.10** | | NADH-dependent fumarate reductase | | | | | | | | | | | | | | | | | | | | - | | - | | | + | - | | - | | | + | | | | - | | | | | | 16 | | | | |
| **Tc00.1047053506297.190** | | pyruvate phosphate dikinase | | | | | | | | | | | | | | | | | | | | 1 | | + | | | + | + | | - | | | + | | | | - | | | | | | 17,18,19 | | | | |
| **Tc00.1047053507883.80** | | adenylate kinase | | | | | | | | | | | | | | | | | | | | 1 | | - | | | + | - | | - | | | + | | | | - | | | | | | 20,21,22 | | | | |
| **Tc00.1047053509733.180** | | adenylate kinase | | | | | | | | | | | | | | | | | | | | 1 | | - | | | - | + | | - | | | + | | | | - | | | | | | 20,21,22 | | | | |
| **Tc00.1047053506947.90** | | adenylate kinase | | | | | | | | | | | | | | | | | | | | - | | - | | | + | - | | - | | | + | | | | - | | | | | | 20,21,22 | | | | |
| **Tc00.1047053503479.30** | | adenylate kinase | | | | | | | | | | | | | | | | | | | | - | | - | | | - | + | | - | | | + | | | | - | | | | | | 20,21,22 | | | | |
| **Tc00.1047053508461.400** | | nucleoside diphosphate kinase | | | | | | | | | | | | | | | | | | | | 2 | | - | | | + | + | | - | | | - | | | | - | | | | | | 23 | | | | |
| **Tc00.1047053507241.30** | | arginine kinase | | | | | | | | | | | | | | | | | | | | 2 | | + | | | - | - | | - | | | - | | | | - | | | | | | 24 | | | | |
|  | | | | | | | | | | | | | | | | | | | | | | | | | | | | | | | | | | | | | | | | | | | | | | | |
| **Pentose-Phosphate Pathway** | | | | | | | | |  | | | | | |  |  | | |  |  | | | |  | |  | | | | | | | | |  | | | | | | |  |  |  |  |  |  |
| **Tc00.1047053506925.480** | | ribokinase | | | | | | | | | | | | | | | | | | | 1 | | | + | | | + | + | | - | | | + | | | | - | | | | | | 25 | | | | |
| **Tc00.1047053508625.150** | | L-ribulokinase | | | | | | | | | | | | | | | | | | | 1 | | | + | | | + | + | | - | | | - | | | | - | | | | | | - | | | | |
| **Tc00.1047053504117.20** | | sedoheptulose-1,7-bisphosphatase | | | | | | | | | | | | | | | | | | | 1 | | | + | | | + | - | | + | | | + | | | | - | | | | | | 9 | | | | |
| **Tc00.1047053507889.10** | | transaldolase | | | | | | | | | | | | | | | | | | | - | | | + | | | - | + | | - | | | - | | | | - | | | | | | 26 | | | | |
| **Tc00.1047053508415.40** | | ADP-ribosylation factor 1 | | | | | | | | | | | | | | | | | | | - | | | - | | | + | - | | + | | | - | | | | - | | | | | | - | | | | |
|  | | | | | | | | | | | | | | | | | | | | | | | | | | | | | | | | | | | | | | | | | | | | | |  |  |
| **Glycerol metabolism** | | | | | | |  | | | | | | | | | | | | | |  | | |  | | |  |  | |  | | |  | | | |  | | | | | |  | | |  |  |
| **Tc00.1047053511151.90** | | glycerol-3-phosphate dehydrogenase | | | | | | | | | | | | | | | | | | | - | | | - | | | + | - | | *+* | | | + | | | | - | | | | | | 28,29 | | | | |
| **Tc00.1047053508241.10** | | glycerol-3-phosphate dehydrogenase, putative | | | | | | | | | | | | | | | | | | | - | | | - | | | - | - | | + | | | + | | | | - | | | | | | 28,29 | | | | |
| **Tc00.1047053510661.60** | | glycerol kinase, glycosomal | | | | | | | | | | | | | | | | | | | 1 | | | + | | | + | + | | *-* | | | + | | | | - | | | | | | 1 | | | | |
| **Tc00.1047053503983.20** | | dihydroxyacetone kinase 1-like | | | | | | | | | | | | | | | | | | | 1 | | | - | | | + | + | | - | | | - | | | | - | | | | | | - | | | | |
|  |  | | | | | | | | | | | | | | | | | | | |  | | |  | | |  |  | |  | | |  | | | |  | | | | | |  | | |  |  |
| **Fatty-acid metabolism** | | | | | | | | | | | | | | | | | | | | |  | | |  | | |  |  | |  | | |  | | | |  | | | | | |  | | |  |  |
| **Tc00.1047053511353.4** | | choline/carnitine O-acyltransferase | | | | | | | | | | | | | | | | | | | 1 | | | - | | | - | - | | + | | | - | | | | - | | | | | | - | | | | |
| **Tc00.1047053509999.90** | | carnitine/choline acetyltransferase | | | | | | | | | | | | | | | | | | | 1 | | | + | | | - | - | | - | | | - | | | | - | | | | | | - | | | | |
| **Tc00.1047053508827.40** | | acyl-CoA dehydrogenase | | | | | | | | | | | | | | | | | | | - | | | - | | | + | + | | - | | | - | | | | - | | | | | | - | | | | |
| **Tc00.1047053507547.40** | | enoyl-CoA hydratase/enoyl-CoA isomerase/3-hydroxyacyl-CoA dehydrogenase | | | | | | | | | | | | | | | | | | | 2 | | | + | | | + | - | | + | | | + | | | | - | | | | | | 30 | | | | |
| **Tc00.1047053508441.70** | | enoyl-CoA hydratase/enoyl-CoA isomerase/3-hydroxyacyl-CoA dehydrogenase | | | | | | | | | | | | | | | | | | | 2 | | | + | | | - | + | | - | | | + | | | | - | | | | | | 30 | | | | |
| **Tc00.1047053509717.90** | | short chain 3-hydroxyacyl-CoA dehydrogenase | | | | | | | | | | | | | | | | | | | 1 | | | + | | | + | + | | - | | | - | | | | - | | | | | | - | | | | |
| **Tc00.1047053506727.100** | | enoyl-CoA hydratase/isomerase family protein | | | | | | | | | | | | | | | | | | | - | | | + | | | - | - | | - | | | - | | | | - | | | | | | 30 | | | | |
| **Tc00.1047053509463.30** | | 3-ketoacyl-CoA thiolase | | | | | | | | | | | | | | | | | | | 1 | | | + | | | + | + | | - | | | - | | | | - | | | | | | 25,31 | | | | |
| **Tc00.1047053504055.40** | | acyltransferase | | | | | | | | | | | | | | | | | | | 1 | | | - | | | + | - | | + | | | - | | | | - | | | | | | - | | | | |
| **Tc00.1047053506435.270** | | acyltransferase | | | | | | | | | | | | | | | | | | | 1 | | | - | | | + | - | | + | | | - | | | | - | | | | | | - | | | | |
| **Tc00.1047053511389.150** | | thiolase protein-like protein | | | | | | | | | | | | | | | | | | | - | | | + | | | + | - | | + | | | - | | | | - | | | | | | 31 | | | | |
| **Tc00.1047053507107.40** | | 3,2-trans-enoyl-CoA isomerase | | | | | | | | | | | | | | | | | | | - | | | - | | | - | + | | - | | | - | | | | - | | | | | | - | | | | |
| **Tc00.1047053503575.50** | | fatty acyl CoA synthetase | | | | | | | | | | | | | | | | | | | - | | | - | | | + | - | | - | | | + | | | | - | | | | | | - | | | | |
| **Tc00.1047053494675.10** | | fatty acyl CoA synthetase 2 | | | | | | | | | | | | | | | | | | | - | | | - | | | + | - | | - | | | - | | | | - | | | | | | - | | | | |
| **Tc00.1047053506661.20** | | fatty acid elongase | | | | | | | | | | | | | | | | | | | - | | | - | | | + | - | | - | | | - | | | | - | | | | | | - | | | | |
| **Tc00.1047053506661.30** | | fatty acid elongase | | | | | | | | | | | | | | | | | | | - | | | - | | | - | - | | + | | | - | | | | - | | | | | | - | | | | |
| **Tc00.1047053510877.55** | | acyl-CoA binding protein | | | | | | | | | | | | | | | | | | | 1 | | | - | | | + | - | | - | | | + | | | | - | | | | | | 49 | | | | |
| **Tc00.1047053506489.20** | | monoglyceride lipase | | | | | | | | | | | | | | | | | | | - | | | - | | | + | - | | + | | | + | | | | - | | | | | | - | | | | |
|  | |  | | | | | | | | | | | | | | | | | | |  | | |  | | |  |  | |  | | |  | | | |  | | | | | |  | | | | |
| **Ether-lipid synthesis** | | | | | | | | | | | | | | | | | | | | | | | | | | | | | | | | | | | | | | | | | | | | | |  |  |
| **Tc00.1047053503815.10** | | alkyl-dihydroxyacetone phosphate synthase | | | | | | | | | | | | | | | | | | | 1 | | | + | | | + | + | | + | | | + | | | | - | | | | | | 32 | | | | |
| **Tc00.1047053505807.110** | | alkyl-dihydroxyacetone phosphate synthase | | | | | | | | | | | | | | | | | | | 1 | | | - | | | + | + | | - | | | + | | | | - | | | | | | 32 | | | | |
|  | |  | | | | | | | | | | | | | | | | | | |  | | |  | | |  |  | |  | | |  | | | |  | | | | | |  | | | | |
| **Sterol synthesis** | | | | | | | | | | | | | | | | | | | | | | | | | | | | | | | | | | | | | | | | | | | | | |  |  |
| **Tc00.1047053511903.40** | | 3-hydroxy-3-methylglutaryl-CoA synthase, putative | | | | | | | | | | | | | | | | | | | - | | | - | | | - |  | | + | | | - | | | | - | | | | | | - | | | | |
| **Tc00.1047053506831.40** | | 3-hydroxy-3-methylglutaryl-CoA reductase | | | | | | | | | | | | | | | | | | | - | | | - | | | + | + | | - | | | - | | | | - | | | | | | 33 | | | | |
| **Tc00.1047053509237.10** | | mevalonate kinase | | | | | | | | | | | | | | | | | | | 1 | | | + | | | + | - | | - | | | + | | | | - | | | | | | 34,35,36 | | | | |
| **Tc00.1047053436521.9** | | mevalonate kinase | | | | | | | | | | | | | | | | | | | - | | | - | | | + | + | | - | | | + | | | | - | | | | | | 34,35,36 | | | | |
| **Tc00.1047053510431.10** | | isopentenyl-diphosphate delta-isomerase | | | | | | | | | | | | | | | | | | | 1 | | | + | | | + | + | | - | | | + | | | | - | | | | | | 9 | | | | |
| **Tc00.1047053408799.19** | | isopentenyl-diphosphate delta-isomerase | | | | | | | | | | | | | | | | | | | - | | | - | | | - | + | | - | | | + | | | | - | | | | | | 9 | | | | |
| **Tc00.1047053509589.20** | | squalene monooxygenase | | | | | | | | | | | | | | | | | | | - | | | - | | | + | - | | - | | | - | | | | - | | | | | | - | | | | |
| **Tc00.1047053506297.260** | | lanosterol 14-alpha-demethylase | | | | | | | | | | | | | | | | | | | - | | | - | | | - | - | | + | | | - | | | | - | | | | | | - | | | | |
| **Tc00.1047053510873.10** | | NAD(P)-dependent steroid dehydrogenase protein (fragment) | | | | | | | | | | | | | | | | | | | - | | | - | | | - | - | | + | | | - | | | | - | | | | | | - | | | | |
| **Tc00.1047053505683.10** | | sterol 24-C-methyltransferase | | | | | | | | | | | | | | | | | | | - | | | + | | | + | - | | + | | | - | | | | - | | | | | | - | | | | |
| **Tc00.1047053510329.90** | | C-8 sterol isomerase | | | | | | | | | | | | | | | | | | | 1 | | | - | | | + | - | | - | | | + | | | | - | | | | | | 9 | | | | |
| **Tc00.1047053507709.90** | | sterol C-24 reductase | | | | | | | | | | | | | | | | | | | - | | | - | | | + | - | | + | | |  | | | | - | | | | | | - | | | | |
| **Tc00.1047053457251.10** | | 3-oxo-5-alpha-steroid 4-dehydrogenase | | | | | | | | | | | | | | | | | | | - | | | + | | | + | - | | + | | | + | | | | - | | | | | | 49 | | | | |
|  | |  | | | | | | | | | | | | | | | | | | |  | | |  | | |  |  | |  | | |  | | | |  | | | | | |  | | | | |
| **Purine and pyrimidine synthesis** | | | | | | | | | | | | | | | | | | | | |  | | |  | | |  |  | |  | | |  | | | |  | | | | | |  | | | | |
| **Tc00.1047053507059.60** | | orotidine-5-phosphate decarboxylase/orotate phosphoribosyltransferase | | | | | | | | | | | | | | | | | | | 1 | | | + | | | + | + | | + | | | - | | | | - | | | | | | 37,38,39 | | | | |
| **Tc00.1047053508373.29** | | orotidine-5-phosphate decarboxylase/orotate phosphoribosyltransferase | | | | | | | | | | | | | | | | | | | - | | | - | | | + | + | | - | | | - | | | | - | | | | | | 37,38,39 | | | | |
| **Tc00.1047053506519.130** | | guanosine monophosphate reductase | | | | | | | | | | | | | | | | | | | 1 | | | + | | | + | + | | - | | | - | | | | - | | | | | | 40 | | | | |
| **Tc00.1047053508909.20** | | guanosine monophosphate reductase | | | | | | | | | | | | | | | | | | | 1 | | | + | | | + | + | | - | | | - | | | | - | | | | | | 40 | | | | |
| **Tc00.1047053506519.130** | | inosine-5'-monophosphate dehydrogenase | | | | | | | | | | | | | | | | | | | 1 | | | + | | | + | + | | - | | | - | | | | - | | | | | | 41 | | | | |
| **Tc00.1047053511301.110** | | inosine-5'-monophosphate dehydrogenase | | | | | | | | | | | | | | | | | | | 1 | | | + | | | + | + | | - | | | + | | | | - | | | | | | 41 | | | | |
| **Tc00.1047053507211.40** | | inosine-5'-monophosphate dehydrogenase | | | | | | | | | | | | | | | | | | | 1 | | | - | | | + | - | | - | | | + | | | | - | | | | | | 41 | | | | |
| **Tc00.1047053508909.20** | | inosine-5'-monophosphate dehydrogenase | | | | | | | | | | | | | | | | | | | 1 | | | + | | | + | + | | - | | | + | | | | - | | | | | | 41 | | | | |
| **Tc00.1047053508207.70** | | adenine phosphoribosyltransferase | | | | | | | | | | | | | | | | | | | 1 | | | + | | | + | + | | - | | | + | | | | - | | | | | | 43 | | | | |
| **Tc00.1047053507519.150** | | adenine phosphoribosyltransferase | | | | | | | | | | | | | | | | | | | 1 | | | + | | | + | + | | - | | | + | | | | - | | | | | | 43 | | | | |
| **Tc00.1047053507519.140** | | adenine phosphoribosyltransferase | | | | | | | | | | | | | | | | | | | 1 | | | + | | | + | + | | - | | | + | | | | - | | | | | | 43 | | | | |
| **Tc00.1047053508207.74** | | adenine phosphoribosyltransferase | | | | | | | | | | | | | | | | | | | 1 | | | + | | | - | - | | - | | | + | | | | - | | | | | | 43 | | | | |
| **Tc00.1047053506457.40** | | hypoxanthine-guanine phosphoribosyltransferase | | | | | | | | | | | | | | | | | | | 1 | | | + | | | + | + | | + | | | + | | | | - | | | | | | 44 | | | | |
| **Tc00.1047053509693.80** | | hypoxanthine-guanine phosphoribosyltransferase | | | | | | | | | | | | | | | | | | | 1 | | | - | | | - | + | | - | | | + | | | | - | | | | | | 44 | | | | |
| **Tc00.1047053509693.70** | | hypoxanthine-guanine phosphoribosyltransferase | | | | | | | | | | | | | | | | | | | 1 | | | - | | | + | - | | - | | | + | | | | - | | | | | | 44 | | | | |
| **Tc00.1047053506457.30** | | hypoxanthine-guanine phosphoribosyltransferase | | | | | | | | | | | | | | | | | | | 1 | | | + | | | + | - | | - | | | + | | | | - | | | | | | 44 | | | | |
| **Tc00.1047053511857.80** | | AMP deaminase | | | | | | | | | | | | | | | | | | | 1 | | | - | | | + | - | | + | | | + | | | | - | | | | | | 25 | | | | |
| **Tc00.1047053509569.100** | | nucleoside phosphorylase | | | | | | | | | | | | | | | | | | | 2 | | | + | | | + | + | | - | | | - | | | | - | | | | | | - | | | | |
| **Tc00.1047053504103.109** | | phosphorybosylpyrophosphate synthetase | | | | | | | | | | | | | | | | | | | 1 | | | + | | | - | + | | + | | | - | | | | - | | | | | | 25 | | | | |
|  | | | | | | | | | | | | | | | | | | | | | | | | | | | | | | | | | | | | | | | | | | | | | | | |
| **Trypanothione synthesis** | | | | | | | | | | | | | | | | | | | | | | | | | | | | | | | | | | | | | | | | | | | | | | | |
| **Tc00.1047053508971.40** | | acetylornithine deacetylase-like | | | | | | | | | | | | | | | | | | | 1 | | | + | | | + | + | | - | | | + | | | | - | | | | | | - | | | | |
| **Tc00.1047053511899.40** | | 2-amino-3-ketobutyrate coenzyme A ligase | | | | | | | | | | | | | | | | | | | - | | | + | | | - | + | | - | | | - | | | | - | | | | | | - | | | | |
| **Tc00.1047053509099.50** | | trypanothione synthetase | | | | | | | | | | | | | | | | | | | - | | | + | | | - | - | | - | | | - | | | | - | | | | | | - | | | | |
|  | | | | | | | | | | | | | | | | | | | | | | | | | | | | | | | | | | | | | | | | | | | | | | | |
| **Antioxidant defense** | | | | |  | | |  | |  | |  | | | | |  | | | |  | | |  | | | | | | | | |  | | | | |  |  |  |  |  |  |  |  |  |  |
| **Tc00.1047053503899.130** | | glutathione peroxidase-like protein | | | | | | | | | | | | | | | | | | | 2 | | | - | | | + | + | | - | | | - | | | | - | | | | | | 45 | | | | |
| **Tc00.1047053508265.10** | | glutathione-S-transferase/glutaredoxin | | | | | | | | | | | | | | | | | | | - | | | - | | | - | - | | + | | | - | | | | - | | | | | | - | | | | |
| **Tc00.1047053506443.70** | | glutathione-S-transferase/glutaredoxin | | | | | | | | | | | | | | | | | | | - | | | - | | | + | - | | + | | | - | | | | - | | | | | | - | | | | |
| **Tc00.1047053507259.10** | | tryparedoxin peroxidase | | | | | | | | | | | | | | | | | | | - | | | + | | | + | + | | - | | | - | | | | - | | | | | | - | | | | |
| **Tc00.1047053508649.5** | | tryparedoxin peroxidase | | | | | | | | | | | | | | | | | | | - | | | + | | | + | + | | - | | | - | | | | - | | | | | | - | | | | |
| **Tc00.1047053484299.10** | | trypanothione reductase | | | | | | | | | | | | | | | | | | | 1 | | | - | | | - | + | | - | | | + | | | | - | | | | | | - | | | | |
| **Tc00.1047053504507.5** | | trypanothione reductase | | | | | | | | | | | | | | | | | | | - | | | + | | | + | - | | - | | | + | | | | - | | | | | | - | | | | |
| **Tc00.1047053509997.30** | | tryparedoxin 1 | | | | | | | | | | | | | | | | | | | 2 | | | + | | | + | + | | - | | | + | | | | - | | | | | | - | | | | |
| **Tc00.1047053511715.10** | | iron superoxide dismutase | | | | | | | | | | | | | | | | | | | - | | | - | | | + | - | | - | | | + | | | | - | | | | | | 46,47 | | | | |
| **Tc00.1047053507061.30** | | iron superoxide dismutase | | | | | | | | | | | | | | | | | | | - | | | - | | | + | - | | - | | | + | | | | - | | | | | | 46,47 | | | | |
| **Tc00.1047053508445.20** | | iron superoxide dismutase | | | | | | | | | | | | | | | | | | | - | | | + | | | - | + | | - | | | + | | | | - | | | | | | 46,47 | | | | |
|  | | | | | | | | | | | | | | | | | | | | | | | | | | | | | | | | | | | | | | | | | | | | | | | |
| **Redox metabolism** | | | | | |  | | | | | | | | | | | | | | |  | | |  | | |  |  | |  | |  | | | |  | | | | | | |  | | | | |
| **Tc00.1047053503873.10** | | cytochrome-B5 reductase | | | | | | | | | | | | | | | | | | | 1 | | | + | | | + | - | | + | | - | | | | - | | | | | | | - | | | | |
| **Tc00.1047053506821.210** | | cytochrome-B5 reductase | | | | | | | | | | | | | | | | | | | 1 | | | - | | | - | - | | + | | - | | | | - | | | | | | | - | | | | |
| **Tc00.1047053508543.140** | | electron transfer flavoprotein-ubiquinone oxidoreductase | | | | | | | | | | | | | | | | | | | 1 | | | - | | | + | - | | + | | - | | | | - | | | | | | | - | | | | |
| **Tc00.1047053508409.160** | | thymine dioxygenase, putative | | | | | | | | | | | | | | | | | | | - | | | - | | | - | + | | - | | - | | | | - | | | | | | | - | | | | |
| **Tc00.1047053505183.30** | | malic enzyme | | | | | | | | | | | | | | | | | | | - | | | + | | | - | + | | - | | - | | | | - | | | | | | | - | | | | |
| **Tc00.1047053508647.280** | | malic enzyme | | | | | | | | | | | | | | | | | | | - | | | + | | | + | - | | - | | - | | | | - | | | | | | | - | | | | |
| **Tc00.1047053505183.20** | | malic enzyme | | | | | | | | | | | | | | | | | | | - | | | + | | | - | - | | - | | - | | | | - | | | | | | | - | | | | |
|  | | | | | | | | | | | | | | | | | | | | | | | | | | | | | | | | | | | | | | | | | | | | | | |  |
| **Amino-acid metabolism** | | | | | | | | | | | | | | | | | | | | | | | | | | | | | | | | | | | | | | | | | | | | | | |  |
|  | |  | | | | | | | | | | | | | | | | | | |  | | |  | | |  |  | |  | |  | | | |  | | | | | | |  | | | |  |
| **Tc00.1047053506247.220** | | histidine ammonia-lyase | | | | | | | | | | | | | | | | | | | 1 | | | + | | | + | + | | + | | - | | | | - | | | | | | | - | | | |  |
| **Tc00.1047053504045.110** | | urocanate hydratase | | | | | | | | | | | | | | | | | | | - | | | + | | | - | + | | - | | - | | | | - | | | | | | | - | | | |  |
| **Tc00.1047053509137.30** | | imidazolonepropionase | | | | | | | | | | | | | | | | | | | 2 | | | + | | | - | - | | - | | - | | | | - | | | | | | | - | | | |  |
| **Tc00.1047053506411.30** | | proline dehydrogenase | | | | | | | | | | | | | | | | | | | 1 | | | - | | | + | - | | + | | - | | | | - | | | | | | | - | | | |  |
| **Tc00.1047053509207.90** | | pyrroline-5-carboxylate reductase | | | | | | | | | | | | | | | | | | | 1 | | | - | | | - | + | | - | | - | | | | - | | | | | | | - | | | |  |
| **Tc00.1047053510943.50** | | delta-1-pyrroline-5-carboxylate dehydrogenase | | | | | | | | | | | | | | | | | | | - | | | + | | | + | + | | - | | - | | | | - | | | | | | | - | | | |  |
| **Tc00.1047053503577.9** | | delta-1-pyrroline-5-carboxylate dehydrogenase | | | | | | | | | | | | | | | | | | | - | | | - | | | - | + | | - | | - | | | | - | | | | | | | - | | | |  |
| **Tc00.1047053507923.10** | | L-threonine 3-dehydrogenase | | | | | | | | | | | | | | | | | | | 1 | | | + | | | - | + | | - | | - | | | | - | | | | | | | - | | | |  |
| **Tc00.1047053506681.70** | | threonine dehydratase-like | | | | | | | | | | | | | | | | | | | 1 | | | + | | | + | - | | - | | - | | | | - | | | | | | | - | | | |  |
| **Tc00.1047053506825.70** | | threonine dehydratase-like | | | | | | | | | | | | | | | | | | | 1 | | | - | | | + | - | | - | | - | | | | - | | | | | | | - | | | |  |
| **Tc00.1047053510889.140** | | alanine aminotransferase | | | | | | | | | | | | | | | | | | | - | | | + | | | + | - | | - | | - | | | | - | | | | | | | - | | | |  |
| **Tc00.1047053506529.430** | | alanine aminotransferase | | | | | | | | | | | | | | | | | | | - | | | + | | | + | + | | - | | - | | | | - | | | | | | | - | | | |  |
| **Tc00.1047053508111.30** | | glutamate dehydrogenase | | | | | | | | | | | | | | | | | | | - | | | + | | | + | + | | - | | - | | | | - | | | | | | | - | | | |  |
| **Tc00.1047053505843.10** | | glutamate dehydrogenase | | | | | | | | | | | | | | | | | | | - | | | + | | | - | + | | - | | - | | | | - | | | | | | | - | | | |  |
| **Tc00.1047053509445.39** | | glutamate dehydrogenase | | | | | | | | | | | | | | | | | | | - | | | + | | | - | + | | - | | - | | | | - | | | | | | | - | | | |  |
| **Tc00.1047053510187.30** | | tyrosine aminotransferase | | | | | | | | | | | | | | | | | | | - | | | - | | | + | - | | - | | - | | | | - | | | | | | | - | | | |  |
| **Tc00.1047053508537.5** | | tyrosine aminotransferase | | | | | | | | | | | | | | | | | | | - | | | - | | | - | + | | - | | - | | | | - | | | | | | | - | | | |  |
| **Tc00.1047053510795.10** | | tyrosine aminotransferase | | | | | | | | | | | | | | | | | | | - | | | - | | | - | + | | - | | - | | | | - | | | | | | | - | | | |  |
| **Tc00.1047053511461.20** | | tyrosine aminotransferase | | | | | | | | | | | | | | | | | | | - | | | + | | | - | - | | - | | - | | | | - | | | | | | | - | | | |  |
| **Tc00.1047053510565.11** | | tyrosine aminotransferase | | | | | | | | | | | | | | | | | | | - | | | - | | | + | - | | - | | - | | | | - | | | | | | | - | | | |  |
| **Tc00.1047053506629.220** | | isovaleryl-coA dehydrogenase | | | | | | | | | | | | | | | | | | | - | | | + | | | - | + | | - | | - | | | | - | | | | | | | - | | | |  |
| **Tc00.1047053506853.50** | | 2-oxoisovalerate dehydrogenase alpha subunit | | | | | | | | | | | | | | | | | | | 1 | | | + | | | + | - | | - | | - | | | | - | | | | | | | - | | | |  |
| **Tc00.1047053511141.20** | | choline dehydrogenase | | | | | | | | | | | | | | | | | | | - | | | - | | | + | - | | - | | - | | | | - | | | | | | | - | | | |  |
| **Tc00.1047053506851.20** | | choline dehydrogenase | | | | | | | | | | | | | | | | | | | - | | | - | | | - | - | | + | | - | | | | - | | | | | | | - | | | |  |
|  | |  | | | | | | | | | | | | | | | | | | |  | | |  | | |  |  | |  | |  | | | | |  | | | | | |  | | | |  |
| **TCA cycle** | | | | | | | | | | | | | | | | | | | | | | | | | | | | | | | | | | | | | | | | | | | | | | |  |
| **Tc00.1047053510351.90** | | dihydrolipoamide branched chain transacylase | | | | | | | | | | | | | | | | | | | 2 | | | - | | | - | + | | - | | - | | | | | - | | | | | | - | | | |  |
| **Tc00.1047053507601.70** | | dihydrolipoamide branched chain transacylase | | | | | | | | | | | | | | | | | | | 2 | | | + | | | - | - | | - | | - | | | | | - | | | | | | - | | | |  |
| **Tc00.1047053506925.319** | | isocitrate dehydrogenase | | | | | | | | | | | | | | | | | | | 1 | | | + | | | + | + | | - | | + | | | | | - | | | | | | 48,49 | | | |  |
|  | |  | | | | | | | | | | | | | | | | | | |  | | |  | | |  |  | |  | | |  | | | | | | | | | |  | | | |  |
| **Proteases** | | | | | | | | | | | | | | | | | | | | | | | | | | | | | | | | | | | | | | | | | | | | | | |  |
| **Tc00.1047053508999.220** | | calpain-like cysteine peptidase | | | | | | | | | | | | | | | | | | | - | | | - | | | + | - | | - | | - | | | | | - | | | | | | - | | | |  |
| **Tc00.1047053509003.30** | | calpain-like cysteine peptidase | | | | | | | | | | | | | | | | | | | - | | | + | | | + | + | | + | | - | | | | | - | | | | | | - | | | |  |
| **Tc00.1047053503855.40** | | calpain-like cysteine peptidase | | | | | | | | | | | | | | | | | | | - | | | - | | | - | - | | + | | - | | | | | - | | | | | | - | | | |  |
| **Tc00.1047053506983.39** | | calpain-like cysteine peptidase | | | | | | | | | | | | | | | | | | | - | | | + | | | - | + | | + | | - | | | | | - | | | | | | - | | | |  |
| **Tc00.1047053484311.10** | | calpain-like cysteine peptidase | | | | | | | | | | | | | | | | | | | - | | | - | | | - | + | | - | | - | | | | | - | | | | | | - | | | |  |
| **Tc00.1047053506721.30** | | calpain-like cysteine peptidase | | | | | | | | | | | | | | | | | | | - | | | + | | | - | + | | - | | - | | | | | - | | | | | | - | | | |  |
| **Tc00.1047053503987.20** | | calpain-like cysteine peptidase | | | | | | | | | | | | | | | | | | | - | | | + | | | + | + | | - | | - | | | | | - | | | | | | - | | | |  |
| **Tc00.1047053509001.40** | | calpain-like cysteine peptidase | | | | | | | | | | | | | | | | | | | - | | | - | | | + | - | | - | | - | | | | | - | | | | | | - | | | |  |
| **Tc00.1047053508555.70** | | calpain-like cysteine peptidase | | | | | | | | | | | | | | | | | | | - | | | + | | | + | + | | + | | - | | | | | - | | | | | | - | | | |  |
| **Tc00.1047053506563.220** | | calpain-like cysteine peptidase | | | | | | | | | | | | | | | | | | | - | | | + | | | - | + | | - | | - | | | | | - | | | | | | - | | | |  |
| **Tc00.1047053504153.160** | | metallo-peptidase | | | | | | | | | | | | | | | | | | | 2 | | | - | | | - | + | | - | | - | | | | | - | | | | | | - | | | |  |
| **Tc00.1047053506513.110** | | peptidase T | | | | | | | | | | | | | | | | | | | 1 | | | + | | | + | + | | + | | - | | | | | - | | | | | | - | | | |  |
| **Tc00.1047053507305.30** | | casein kinase | | | | | | | | | | | | | | | | | | | 2 | | | - | | | + | + | | - | | - | | | | | - | | | | | | - | | | |  |
| **Tc00.1047053507689.30** | | metallo-peptidase, clan MH, family M18, putative | | | | | | | | | | | | | | | | | | | 1 | | | - | | | + | - | | + | | - | | | | | - | | | | | | - | | | |  |
| **Tc00.1047053507689.40** | | metallo-peptidase, clan MH, family M18, putative | | | | | | | | | | | | | | | | | | | 1 | | | + | | | + | + | | + | | - | | | | | - | | | | | | - | | | |  |
| **Tc00.1047053507689.50** | | metallo-peptidase, clan MH, family M18, putative (fragment) | | | | | | | | | | | | | | | | | | | 1 | | | + | | | + | + | | - | | - | | | | | - | | | | | | - | | | |  |
| **Tc00.1047053507657.20** | | metallo-peptidase, clan MH, family M18, putative | | | | | | | | | | | | | | | | | | | 1 | | | + | | | + | + | | - | | *-* | | | | | - | | | | | | - | | | |  |
| **Tc00.1047053511391.120** | | metallo-peptidase, clan MH, family M18, putative | | | | | | | | | | | | | | | | | | | 1 | | | + | | | + | + | | + | | *-* | | | | | - | | | | | | - | | | |  |
| **Tc00.1047053510837.20** | | metallo-peptidase, clan MH, family M18, putative | | | | | | | | | | | | | | | | | | | 1 | | | + | | | + | + | | + | | *-* | | | | | - | | | | | | - | | | |  |
|  | |  | | | | | | | | | | | | | | | | | | |  | | |  | | |  |  | |  |  | | | | | | | | | |  | |  | | | |  |
| **Transporters/integral membrane proteins** | | | | | | | | | | | | | | | | | | | | |  | | |  | | |  |  | |  |  | | | | | | | | | |  | |  | | | |  |
| **Tc00.1047053508479.230** | | PEX2 | | | | | | | | | | | | | | | | | | | - | | | + | | | + | - | | + | | + | | | | | - | | | | | | 49 | | | |  |
| **Tc00.1047053503809.20** | | peroxisome assembly protein (PEX12) | | | | | | | | | | | | | | | | | | | - | | | - | | | + | - | | + | | + | | | | | - | | | | | | 49,55 | | | |  |
| **Tc00.1047053508479.190** | | peroxisome assembly protein (PEX10) | | | | | | | | | | | | | | | | | | | 1 | | | - | | | + | - | | + | | + | | | | | - | | | | | | 55 | | | |  |
| **Tc00.1047053511145.40** | | PEX14 | | | | | | | | | | | | | | | | | | | - | | | - | | | + | + | | - | | + | | | | | - | | | | | | 49 | | | |  |
| **Tc00.1047053507009.10** | | GIM5A | | | | | | | | | | | | | | | | | | | - | | | - | | | + | - | | - | | + | | | | | - | | | | | | 49 | | | |  |
| **Tc00.1047053510669.20** | | GIM5A | | | | | | | | | | | | | | | | | | | - | | | + | | | + | - | | + | | + | | | | | - | | | | | | 49 | | | |  |
| **Tc00.1047053508613.30** | | peroxisomal membrane protein 4 | | | | | | | | | | | | | | | | | | | 1 | | | - | | | + | - | | + | | + | | | | | - | | | | | | - | | | |  |
| **Tc00.1047053510043.40** | | peroxisomal membrane protein (PEX16), putative | | | | | | | | | | | | | | | | | | | - | | | - | | | - | - | | + | | - | | | | | - | | | | | | 50 | | | |  |
| **Tc00.1047053504005.40** | | PEX11 | | | | | | | | | | | | | | | | | | | - | | | - | | | - | - | | + | | - | | | | | - | | | | | | 49 | | | |  |
| **Tc00.1047053503811.40** | | peroxisomal targeting signal type 2 receptor (PEX7) | | | | | | | | | | | | | | | | | | | - | | | - | | | + | - | | - | | + | | | | | - | | | | | | - | | | |  |
| **Tc00.1047053509877.50** | | glycosomal membrane protein (PEX11) | | | | | | | | | | | | | | | | | | | - | | | - | | | + | - | | + | | + | | | | | - | | | | | | 49 | | | |  |
| **Tc00.1047053511017.50** | | glycosomal membrane protein (PEX11) | | | | | | | | | | | | | | | | | | | - | | | + | | | + | - | | + | | + | | | | | - | | | | | | 49 | | | |  |
| **Tc00.1047053503833.30** | | putative PEX13 | | | | | | | | | | | | | | | | | | | 1 | | | - | | | + | - | | - | | + | | | | | - | | | | | | 51,52 | | | |  |
| **Tc00.1047053507093.260** | | ABC transporter, mitochondrial, putative | | | | | | | | | | | | | | | | | | | - | | | + | | | - | - | | + | | - | | | | | - | | | | | | - | | | |  |
| **Tc00.1047053506249.70** | | ABC transporter | | | | | | | | | | | | | | | | | | | 1 | | | - | | | + | - | | + | | - | | | | | - | | | | | | - | | | |  |
| **Tc00.1047053506925.530** | | ABC transporter (GAT3) | | | | | | | | | | | | | | | | | | | - | | | - | | | + | - | | + | | - | | | | | - | | | | | | 53,54 | | | |  |
| **Tc00.1047053510431.150** | | ABC transporter (GAT2) | | | | | | | | | | | | | | | | | | | - | | | - | | | - | - | | + | | - | | | | | - | | | | | | 53,54 | | | |  |
| **Tc00.1047053506579.10** | | ABC transporter | | | | | | | | | | | | | | | | | | | - | | | - | | | - | - | | + | | - | | | | | - | | | | | | - | | | |  |
| **Tc00.1047053506989.30** | | ABC transporter | | | | | | | | | | | | | | | | | | | 2 | | | + | | | - | - | | + | | - | | | | | - | | | | | | - | | | |  |
| **Tc00.1047053507241.39** | | ABC transporter | | | | | | | | | | | | | | | | | | | - | | | - | | | + | + | | - | | - | | | | | - | | | | | | - | | | |  |
| **Tc00.1047053508231.190** | | ABC transporter | | | | | | | | | | | | | | | | | | | 1 | | | - | | | - | - | | + | | - | | | | | - | | | | | | - | | | |  |
| **Tc00.1047053504867.20** | | ABC transporter | | | | | | | | | | | | | | | | | | | - | | | - | | | - | - | | + | | - | | | | | - | | | | | | - | | | |  |
| **Tc00.1047053511537.8** | | ABC transporter of the mitochondrion | | | | | | | | | | | | | | | | | | | - | | | - | | | - | - | | + | | - | | | | | - | | | | | | - | | | |  |
| **Tc00.1047053506025.4** | | membrane-bound acid phosphatase | | | | | | | | | | | | | | | | | | | - | | | - | | | + | - | | + | | - | | | | | - | | | | | | - | | | |  |
| **Tc00.1047053511209.69** | | membrane-bound acid phosphatase | | | | | | | | | | | | | | | | | | | - | | | + | | | + | - | | + | | - | | | | | - | | | | | | - | | | |  |
| **Tc00.1047053508827.10** | | membrane-bound acid phosphatase | | | | | | | | | | | | | | | | | | | - | | | - | | | + | - | | + | | - | | | | | - | | | | | | - | | | |  |
| **Tc00.1047053510649.24** | | membrane associated protein (partial) | | | | | | | | | | | | | | | | | | | - | | | - | | | + | - | | - | | - | | | | | - | | | | | | - | | | |  |
| **Tc00.1047053508551.39** | | hexose transporter (partial) | | | | | | | | | | | | | | | | | | | - | | | - | | | + | - | | - | | - | | | | | - | | | | | | 56 | | | |  |
| **Tc00.1047053506355.10** | | hexose transporter | | | | | | | | | | | | | | | | | | | - | | | - | | | - | - | | + | | - | | | | | - | | | | | | 56 | | | |  |
| **Tc00.1047053511583.40** | | tricarboxylate carrier | | | | | | | | | | | | | | | | | | | - | | | - | | | + | - | | + | | - | | | | | - | | | | | | - | | | |  |
| **Tc00.1047053510581.20** | | receptor-type adenylate cyclase | | | | | | | | | | | | | | | | | | | - | | | - | | | + | - | | + | | - | | | | | - | | | | | | - | | | |  |
| **Tc00.1047053510581.9** | | receptor-type adenylate cyclase | | | | | | | | | | | | | | | | | | | - | | | - | | | - | - | | + | | - | | | | | - | | | | | | - | | | |  |
| **Tc00.1047053507467.10** | | receptor-type adenylate cyclase | | | | | | | | | | | | | | | | | | | - | | | - | | | - | - | | + | | - | | | | | - | | | | | | - | | | |  |
| **Tc00.1047053508415.40** | | ADP-ribosylation factor 1 | | | | | | | | | | | | | | | | | | | - | | | - | | | + | - | | + | | - | | | | | - | | | | | | - | | | |  |
| **Tc00.1047053511249.10** | | ADP/ATP mitochondrial carrier protein, putative | | | | | | | | | | | | | | | | | | | - | | | - | | | + | - | | + | | + | | | | | - | | | | | | - | | | |  |
| **Tc00.1047053509551.30** | | mitochondrial phosphate transporter, putative | | | | | | | | | | | | | | | | | | | - | | | - | | | + | - | | + | | + | | | | | - | | | | | | - | | | |  |
| **Tc00.1047053506657.40** | | ADP/ATP translocase | | | | | | | | | | | | | | | | | | | - | | | - | | | - | - | | + | | - | | | | | - | | | | | | - | | | |  |
| **Tc00.1047053506773.90** | | amino acid permease/transporter | | | | | | | | | | | | | | | | | | | - | | | - | | | + | - | | + | | - | | | | | - | | | | | | - | | | |  |
| **Tc00.1047053506153.10** | | amino acid transporter | | | | | | | | | | | | | | | | | | | - | | | - | | | - | - | | + | | - | | | | | - | | | | | | - | | | |  |
| **Tc00.1047053507585.10** | | amino acid transporter | | | | | | | | | | | | | | | | | | | - | | | - | | | - | - | | + | | - | | | | | - | | | | | | - | | | |  |
| **Tc00.1047053509197.20** | | cation transporter | | | | | | | | | | | | | | | | | | | - | | | - | | | + | - | | + | | - | | | | | - | | | | | | - | | | |  |
| **Tc00.1047053508699.130** | | cation transporter | | | | | | | | | | | | | | | | | | | - | | | - | | | - | - | | + | | - | | | | | - | | | | | | - | | | |  |
|  | |  | | | | | | | | | | | | | | | | | | |  | | |  | | |  |  | |  |  |  | | | | | | | | | | |  | | | |  |
| **Other proteins with PTS or homologs of *T. brucei* proteins detected in glycosomes by Güther *et al.* (2014)** | | | | | | | | | | | | | | | | | | | | | | | | | | | | | | | | | | | | | | | | | | | | | | |  |
| **Tc00.1047053506181.104** | | acetyltransferase-like protein | | | | | | | | | | | | | | | | | | | 1 | | | - | | | + | - | | - | | | + | | | | - | | | | | | 49 | | | |  |
| **Tc00.1047053506999.90** | | Rieske iron-sulfur protein (RISP), mitochondrial precursor | | | | | | | | | | | | | | | | | | | 1 | | | - | | | - | - | | + | | | - | | | | - | | | | | | - | | | |  |
| **Tc00.1047053510759.120** | | Rieske iron-sulfur protein (RISP), mitochondrial precursor | | | | | | | | | | | | | | | | | | | 1 | | | - | | | + | - | | - | | | - | | | | - | | | | | | - | | | |  |
| **Tc00.1047053503419.30** | | thiol-dependent reductase 1 | | | | | | | | | | | | | | | | | | | 2 | | | + | | | - | + | | - | | | - | | | | - | | | | | | - | | | |  |
| **Tc00.1047053509105.70** | | thiol-dependent reductase 1 | | | | | | | | | | | | | | | | | | | 2 | | | + | | | + | - | | - | | | - | | | | - | | | | | | - | | | |  |
| **Tc00.1047053511287.49** | | aldo-keto reductase (AKR) | | | | | | | | | | | | | | | | | | | 1 | | | + | | | + | - | | - | | | - | | | | - | | | | | | - | | | |  |
| **Tc00.1047053511383.50** | | prohibitin | | | | | | | | | | | | | | | | | | | - | | | + | | | + | - | | + | | | - | | | | - | | | | | | - | | | |  |
| **Tc00.1047053511469.70** | | prohibitin | | | | | | | | | | | | | | | | | | | 1 | | | - | | | + | - | | + | | | - | | | | - | | | | | | - | | | |  |
| **Tc00.1047053506295.130** | | prohibitin | | | | | | | | | | | | | | | | | | | 1 | | | - | | | - | - | | + | | | - | | | | - | | | | | | - | | | |  |
| **Tc00.1047053506989.190** | | heat-shock protein 90 | | | | | | | | | | | | | | | | | | | 1 | | | - | | | + | - | | - | | | - | | | | - | | | | | | - | | | |  |
| **Tc00.1047053506843.40** | | retrotransposon hot-spot protein | | | | | | | | | | | | | | | | | | | 1 | | | - | | | - | + | | - | | | + | | | | - | | | | | | - | | | |  |
| **Tc00.1047053503399.10** | | hydrolase-like protein | | | | | | | | | | | | | | | | | | | 1 | | | - | | | + | + | | - | | | - | | | | - | | | | | |  | | | |  |
| **Tc00.1047053506829.80** | | nicotinamidase | | | | | | | | | | | | | | | | | | | 1 | | | - | | | - | + | | - | | | - | | | | - | | | | | | - | | | |  |
| **Tc00.1047053505945.90** | | folate/pteridine transporter | | | | | | | | | | | | | | | | | | | 1 | | | - | | | - | - | | + | | | - | | | | - | | | | | | - | | | |  |
| **Tc00.1047053510381.60** | | proliferator-activated receptor-interacting protein (PRIP) interacting protein (PIMT) | | | | | | | | | | | | | | | | | | | 2 | | | - | | | - | - | | + | | | - | | | | - | | | | | | - | | | |  |
| **Tc00.1047053510955.40** | | axoneme central apparatus protein | | | | | | | | | | | | | | | | | | | 1 | | | - | | | + | + | | - | | | - | | | | - | | | | | | - | | | |  |
| **Tc00.1047053506337.4** | | short-chain dehydrogenase | | | | | | | | | | | | | | | | | | | - | | | - | | | - | - | | + | | | *+* | | | | - | | | | | | - | | | |  |
| **Tc00.1047053511211.160** | | heat-shock protein 70 (HSP70) | | | | | | | | | | | | | | | | | | | - | | | - | | | + | + | | - | | | + | | | | - | | | | | | - | | | |  |
| **Tc00.1047053510439.61** | | heat-shock protein 70(HSP70) | | | | | | | | | | | | | | | | | | | - | | | + | | | - | - | | - | | | + | | | | - | | | | | | - | | | |  |

The data were collected on the Orbitrap Elite MS. The proteins were grouped according to metabolic pathways. TriTrypDB accession number, proteins descriptions are indicated. Presence of PTS1 or PTS2 sequences are indicated as 1 or 2, respectively. The symbols + or - indicate the presence or absence in the supernatants or pellets of the respective treatments. Abbreviations used are: Osm Shock, treatment of glycosomes with osmotic shock; Na_2_CO_3_, treatment of glycosomes with Na_2_CO_3_; sup, supernatant; Güther***,** proteins detected in proteomic analysis as glycosomal with high confidence (Güther et al., 2014); Vertommen**, proteins detected in proteomic analysis as glycosomal (Vertommen et al., 2008).

**References**

1. Opperdoes FR, Borst P. Localization of nine glycolytic enzymes in a microbody-like organelle in *Trypanosoma brucei*: the glycosome. FEBS Lett. 1977 Aug 15;80(2):360-4.
2. Cáceres AJ, Quiñones W, Gualdrón M, Cordeiro A, Avilán L, Michels PA, Concepción JL. Molecular and biochemical characterization of novel glucokinases from *Trypanosoma cruzi* and *Leishmania spp*. Mol Biochem Parasitol. 2007 Dec;156(2):235-45.
3. Taylor MB, Gutteridge WE. *Trypanosoma cruzi*: subcellular distribution of glycolytic and some related enzymes of epimastigotes. Exp Parasitol. 1987 Feb;63(1):84-97.
4. Mottram JC, Coombs GH. *Leishmania mexicana*: subcellular distribution of enzymes in amastigotes and promastigotes. Exp Parasitol. 1985 Jun;59(3):265
5. Concepcion JL, Chataing B, Dubourdieu M. Purification and properties of phosphoglucose isomerases of *Trypanosoma cruzi*. Comp Biochem Physiol B Biochem Mol Biol. 1999 Feb;122(2):211-22
6. Concepción JL, Adjé CA, Quiñones W, Chevalier N, Dubourdieu M, Michels PA. The expression and intracellular distribution of phosphoglycerate kinase isoenzymes in *Trypanosoma cruzi*. Mol Biochem Parasitol. 2001 Nov;118(1):111-21.
7. Barros-Álvarez X, Cáceres AJ, Ruiz MT, Michels PA, Concepción JL, Quiñones W. The glycosomal-membrane associated phosphoglycerate kinase isoenzyme A plays a role in sustaining the glucose flux in *Trypanosoma cruzi* epimastigotes. Mol Biochem Parasitol. 2015 Mar-Apr;200(1-2):5-8.
8. Taylor MB, Gutteridge WE. The occurrence and distribution of alpha-hydroxy-acid dehydrogenase in some members of the order Kinetoplastida. FEBS Lett. 1986 Apr 21;199(2):237-41.
9. Opperdoes FR, Szikora JP. In silico prediction of the glycosomal enzymes of *Leishmania major* and trypanosomes. Mol Biochem Parasitol. 2006 Jun;147(2):193-206.
10. Lobo-Rojas ÁE, González-Marcano EB, Valera-Vera EA, Acosta HR, Quiñones WA, Burchmore RJ, Concepción JL, Cáceres AJ. *Trypanosoma cruzi* contains two galactokinases; molecular and biochemical characterization. Parasitol Int. 2016 Oct;65(5 Pt A):472-82.
11. Bandini G, Mariño K, Güther ML, Wernimont AK, Kuettel S, Qiu W, Afzal S, Kelner A, Hui R, Ferguson MA. Phosphoglucomutase is absent in *Trypanosoma brucei* and redundantly substituted by phosphomannomutase and phospho-N-acetylglucosamine mutase. Mol Microbiol. 2012 Aug;85(3):513-34.
12. Kuettel S, Wadum MC, Güther ML, Mariño K, Riemer C, Ferguson MA. The de novo and salvage pathways of GDP-mannose biosynthesis are both sufficient for the growth of bloodstream-form *Trypanosoma brucei*. Mol Microbiol. 2012 Apr;84(2):340-51.
13. Broman K, Knupfer AL, Ropars M, Deshusses J. Occurrence and role of phosphoenolpyruvate carboxykinase in procyclic *Trypanosoma brucei brucei* glycosomes. Mol Biochem Parasitol. 1983 May;8(1):79-87.
14. Cannata JJ, Valle E, Docampo R, Cazzulo JJ. Subcellular localization of phosphoenolpyruvate carboxykinase in the trypanosomatids *Trypanosoma cruzi* and *Crithidia fasciculata*. Mol Biochem Parasitol. 1982 Sep;6(3):151-60.
15. Cannata JJ, Cazzulo JJ. Glycosomal and mitochondrial malate dehydrogenases in epimastigotes of *Trypanosoma cruzi*. Mol Biochem Parasitol. 1984 Apr;11:37-49.
16. Besteiro S, Biran M, Biteau N, Coustou V, Baltz T, Canioni P, Bringaud F. Succinate secreted by *Trypanosoma brucei* is produced by a novel and unique glycosomal enzyme, NADH-dependent fumarate reductase. J Biol Chem. 2002 Oct 11;277(41):38001-12.
17. Bringaud F1, Baltz D, Baltz T. Functional and molecular characterization of a glycosomal PPi-dependent enzyme in trypanosomatids: pyruvate, phosphate dikinase. Proc Natl Acad Sci U S A. 1998 Jul 7;95(14):7963-8.
18. Acosta H, Dubourdieu M, Quiñones W, Cáceres A, Bringaud F, Concepción JL. Pyruvate phosphate dikinase and pyrophosphate metabolism in the glycosome of *Trypanosoma cruzi* epimastigotes. Comp Biochem Physiol B Biochem Mol Biol. 2004 Aug;138(4):347-56.
19. González-Marcano E, Mijares A, Quiñones W, Cáceres A, Concepción JL. Post-translational modification of the pyruvate phosphate dikinase from *Trypanosoma cruzi*. Parasitol Int. 2014 Feb;63(1):80-6.
20. Opperdoes FR, Markoŝ A, Steiger RF. Localization of malate dehydrogenase, adenylate kinase and glycolytic enzymes in glycosomes and the threonine pathway in the mitochondrion of cultured procyclic trypomastigotes of *Trypanosoma brucei*. Mol Biochem Parasitol. 1981 Dec 31;4(5-6):291-309.
21. Ginger ML, Ngazoa ES, Pereira CA, Pullen TJ, Kabiri M, Becker K, Gull K, Steverding D. Intracellular positioning of isoforms explains an unusually large adenylate kinase gene family in the parasite *Trypanosoma brucei*. J Biol Chem. 2005 Mar 25;280(12):11781-9.
22. Milagros Camara Mde L, Bouvier LA, Miranda MR, Pereira CA. Identification and validation of *Trypanosoma cruzi*'s glycosomal adenylate kinase containing a peroxisomal targeting signal. Exp Parasitol. 2012 Apr;130(4):408-11.
23. Los Milagros Camara M, Bouvier L, Reigada C, Digirolamo FA, Saye M, Pereira CA. A novel stage-specific glycosomal nucleoside diphosphate kinase from *Trypanosoma cruzi*. Folia Parasitol (Praha). 2017 Feb 17;64. pii: 2017.006.
24. Voncken F, Gao F, Wadforth C, Harley M, Colasante C. The phosphoarginine energy-buffering system of *Trypanosoma brucei* involves multiple arginine kinase isoforms with different subcellular locations. PLoS One. 2013; 8(6): e65908.
25. Kerkhoven EJ, Achcar F, Alibu VP, Burchmore RJ, Gilbert IH, Trybiło M, Driessen NN, Gilbert D, Breitling R, Bakker BM, Barrett MP. Handling uncertainty in dynamic models: the pentose phosphate pathway in *Trypanosoma brucei*. PLoS Comput Biol. 2013;9(12):e1003371.
26. Maugeri DA, Cazzulo JJ. The pentose phosphate pathway in *Trypanosoma cruzi*. FEMS Microbiol Lett. 2004 May 1;234(1):117-23.
27. Naderer T, Ellis MA, Sernee MF, De Souza DP, Curtis J, Handman E, McConville MJ. Virulence of *Leishmania major* in macrophages and mice requires the gluconeogenic enzyme fructose-1,6-bisphosphatase. Proc Natl Acad Sci U S A. 2006 Apr 4;103(14):5502-7.
28. Opperdoes FR, Borst P, Bakker S, Leene W. Localization of glycerol-3-phosphate oxidase in the mitochondrion and particulate NAD^+^-linked glycerol-3-phosphate dehydrogenase in the microbodies of the bloodstream form to *Trypanosoma brucei*. Eur J Biochem. 1977 Jun 1;76(1):29-39.
29. Concepcion JL, Acosta H, Quiñones W, Dubourdieu M. A alpha-glycerophosphate dehydrogenase is present in *Trypanosoma cruzi* glycosomes. Mem Inst Oswaldo Cruz. 2001 Jul;96(5):697-701.
30. Wiemer, E, IJlst L, van Roy J, Wanders R, Opperdoes FR. Identification of 2-enoyl coenzyme A hydratase and NADP^+^-dependent 3-hydroxyacyl-CoA dehydrogenase activity in glycosomes of procyclic *Trypanosoma brucei*. 1996; Mol Biochem Parasitol. 82(1): 107-111.
31. Mazet M, Harijan RK, Kiema TR, Haapalainen AM, Morand P, Morales J, Bringaud F, Wierenga RK, Michels PA. The characterization and evolutionary relationships of a trypanosomal thiolase. Int J Parasitol. 2011 Oct;41(12):1273-83.
32. Zomer AW, Opperdoes FR, van den Bosch H. Alkyl dihydroxyacetone phosphate synthase in glycosomes of *Trypanosoma brucei*. Biochim Biophys Acta. 1995 Jul 13;1257(2):167-73.
33. Concepcion JL, Gonzalez-Pacanowska D, Urbina JA. 3-Hydroxy-3-methyl-glutaryl-CoA reductase in *Trypanosoma (Schizotrypanum) cruzi*: subcellular localization and kinetic properties. Arch Biochem Biophys. 1998;352(1):114-20.
34. Carrero-Lerida J, Perez-Moreno G, Castillo-Acosta VM, Ruiz-Perez LM, Gonzalez-Pacanowska D. Intracellular location of the early steps of the isoprenoid biosynthetic pathway in the Trypanosomatids Leishmania major and Trypanosoma brucei. Int. J. Parasitol. 2009.39, 307–314.
35. Bahia D. A new trick for a conserved enzyme: mevalonate kinase, a glycosomal enzyme, can be secreted by *Trypanosoma cruzi* and modulate cell invasion and signaling. Is it another moonlighting enzyme? Front Cell Infect Microbiol. 2017 Sep 29;7:426.
36. Ferreira ÉR, Horjales E, Bonfim-Melo A, Cortez C, da Silva CV, De Groote M, Sobreira TJ, Cruz MC, Lima FM, Cordero EM, Yoshida N, da Silveira JF, Mortara RA, Bahia D. Unique behavior of *Trypanosoma cruzi* mevalonate kinase: A conserved glycosomal enzyme involved in host cell invasion and signaling. Sci Rep. 2016 Apr 26;6:24610.
37. Hammond DJ, Gutteridge WE, Opperdoes FR. A novel location for two enzymes of de novo pyrimidine biosynthesis in trypanosomes and *Leishmania*. FEBS Lett. 1981 Jun 1;128(1):27-9.
38. Hammond DJ, Gutteridge WE. Studies on the glycosomal orotate phosphoribosyl transferase of *Trypanosoma cruzi*. Mol Biochem Parasitol. 1983 Apr;7(4):319-30.
39. French JB, Yates PA, Soysa DR, Boitz JM, Carter NS, Chang B, Ullman B, Ealick SE. The *Leishmania donovani* UMP synthase is essential for promastigote viability and has an unusual tetrameric structure that exhibits substrate-controlled oligomerization. J Biol Chem. 2011 Jun 10;286(23):20930-41.
40. Bessho T, Okada T, Kimura C, Shinohara T, Tomiyama A, Imamura A, Kuwamura M, Nishimura K, Fujimori K, Shuto S, Ishibashi O, Kubata BK, Inui T. Novel characteristics of *Trypanosoma brucei* guanosine 5'-monophosphate reductase distinct from host animals. PLoS Negl Trop Dis. 2016 Jan 5;10(1):e0004339.
41. Dobie F, Berg A, Boitz JM, Jardim A. Kinetic characterization of inosine monophosphate dehydrogenase of *Leishmania donovani*. Mol Biochem Parasitol. 2007 Mar;152(1):11-21.
42. Zarella-Boitz JM, Rager N, Jardim A, Ullman B. Subcellular localization of adenine and xanthine phosphoribosyl transferases in *Leishmania donovani*. Mol Biochem Parasitol. 2004 Mar;134(1):43-51.
43. Lüscher A, Lamprea-Burgunder E, Graf FE, de Koning HP, Mäser P. *Trypanosoma brucei* adenine-phosphoribosyltransferases mediate adenine salvage and aminopurinol susceptibility but not adenine toxicity. Int J Parasitol Drugs Drug Resist. 2013 Dec 19;4(1):55-63.
44. Shih S, Stenberg P, Ullman B. Immunolocalization of *Trypanosoma brucei* hypoxanthine-guanine phosphoribosyltransferase to the glycosome. Mol Biochem Parasitol. 1998 May 1;92(2):367-71.
45. Schlecker T, Schmidt A, Dirdjaja N, Voncken F, Clayton C, Krauth-Siegel RL. Substrate specificity, localization, and essential role of the glutathione peroxidase-type tryparedoxin peroxidases in *Trypanosoma brucei*. J Biol Chem. 2005 Apr 15;280(15):14385-94.
46. Dey R, Datta SC. Leishmanial glycosomes contain superoxide dismutase. Biochem J. 1994 Jul 15;301 ( Pt 2):317-9.
47. Plewes KA, Barr SD, Gedamu L. Iron superoxide dismutases targeted to the glycosomes of *Leishmania chagasi* are important for survival. Infect Immun. 2003 Oct;71(10):5910-20.
48. Wang X, Inaoka DK, Shiba T, Balogun EO, Allmann S, Watanabe YI, Boshart M, Kita K, Harada S. Expression, purification, and crystallization of type 1 isocitrate dehydrogenase from *Trypanosoma brucei brucei*. Protein Expr Purif. 2017 Oct;138:56-62.
49. Colasante C, Ellis M, Ruppert T, Voncken F. Comparative proteomics of glycosomes from bloodstream form and procyclic culture form *Trypanosoma brucei brucei*. Proteomics 2006.6: 3275-3293.
50. Kalel VC, Schliebs W, Erdmann R. Identification and functional characterization of *Trypanosoma brucei* peroxin 16.Biochim Biophys Acta. 2015 Oct;1853(10 Pt A):2326-37.
51. Brennand A, Rigden DJ, Michels PA. Trypanosomes contain two highly different isoforms of peroxin PEX13 involved in glycosome biogenesis. FEBS Lett. 2012 Jun 21;586(13):1765-71.
52. Verplaetse E, Rigden DJ, Michels PA. Identification, characterization and essentiality of the unusual peroxin 13 from *Trypanosoma brucei*. Biochim Biophys Acta. 2009 Mar;1793(3):516-27.
53. Igoillo-Esteve M, Mazet M, Deumer G, Wallemacq P, Michels PA. Glycosomal ABC transporters of *Trypanosoma brucei*: characterization of their expression, topology and substrate specificity. Int J Parasitol. 2011 Mar;41(3-4):429-38.
54. Colasante C, Voncken F, Manful T, Ruppert T, Tielens AG, van Hellemond JJ, Clayton C. Proteins and lipids of glycosomal membranes from *Leishmania tarentolae* and *Trypanosoma brucei*. F1000 Res. 2013 Jan 29;2:27.
55. Krazy H, Michels PA. Identification and characterization of three peroxins-PEX6, PEX10 and PEX12-involved in glycosome biogenesis in *Trypanosoma brucei*. Biochim Biophys Acta. 2006 Jan;1763(1):6-17.
56. Silber AM, Tonelli RR, Lopes CG, Cunha-e-Silva N, Torrecilhas AC, Schumacher RI, Colli W, Alves MJ. Glucose uptake in the mammalian stages of *Trypanosoma cruzi*. Mol Biochem Parasitol. 2009 Nov;168(1):102-8.

**Table SII.** **Hypothetical proteins detected by mass spectrometry after treatment of *T. cruzi* glycosomes with sodium carbonate.**

Data are compared with the high-confidence *T. brucei* glycosomal protein reported by Güther *et al*. (2014).

| **Accession Number** | **Description** | | | **MW** | **PTS** | **Position**  **PTS**  **(residues)** | **Na_2_CO_3_ pellet** | **Detected in *T. brucei* glycosomes**  **(Güther et al., 2014)** | **TMpred/**  **HMMTOP**  **Significant score** |
| --- | --- | --- | --- | --- | --- | --- | --- | --- | --- |
| **Carbohydrate metabolism** | | | |  |  |  |  |  |  |
| Tc00.1047053508741.170 | glycerate kinase putative | | | 56.877 | AHL | 523-525 | + | - | no |
| Tc00.1047053509179.100 | L-galactonolactone oxidase | | | 57.788 | SHL | 503-505 | + | Tb927.5.2650 | no |
| Tc00.1047053511759.30 | GlcNAc /glycosyltransferase (GlcNAc) | | | 55.734 | - | - | + | - | yes |
| Tc00.1047053509747.40 | macro domain, a high-affinity ADP-ribose binding module found in a variety of proteins as a stand-alone domain or in combination with other domains like in histone macroH2A and some PARPs (poly ADP-ribose polymerases). | | | 30.873 | - | - | + | Tb11.01.6715 | yes |
| Tc00.1047053511003.190 | D-hexose-6-phosphate epimerase-like | | | 32.683 | SNL | 289-291 | - | Tb927.4.1360 | no |
| Tc00.1047053507897.4 | a superfamily of metal-dependent phosphatases with various substrates/ fructose-1,6-bisphospatase, hydrolyzes fructose 1,6,-bisphosphate to fructose 6-phosphate | | | 42.959 | SSL | 387-389 | - | - | yes |
| **Lipid metabolism** | |  | |  |  |  |  |  |  |
| Tc00.1047053508927.20 | glycosomal ABC transporter, putative (GAT1) | | | 76.570 | YDV | 671-674 |  | - |  |
| Tc00.1047053510877.55 | acyl-CoA binding protein, putative | | | 10.804 | SKL | 91-93 |  | Tb927.4.2010 |  |
| Tc00.1047053507257.150 | alpha/beta-hydrolases | | | 50.605 | SKL | 442-445 |  | - |  |
| Tc00.1047053504425.70 | ABC transporter | | | 130.885 | - | - |  | - |  |
| Tc00.1047053503399.10 | esterase-lipase /esterases and lipases | | | 44.634 | SRL | 404-406 |  | - |  |
| Tc00.1047053510721.10 | fatty acyl CoA reductases (FARs), extended (e) SDRs/putative NAD(P) binding site | | | 57.704 | SSL | 506-508 |  | - |  |
| Tc00.1047053511909.10 | fatty acyl CoA reductases (FARs), extended (e) SDRs /putative NAD(P) binding site | | | 67.824 | SSL | 595-597 |  | - |  |
| Tc00.1047053509455.114 | protein of unknown function (DUF1295), putative (POMP23) | | | 29.022 | - | - |  | - |  |
| Tc00.1047053511901.30 | PI-PLCc-GDPD-SF/ catalytic domain of phosphoinositide-specific phospholipase C-like phosphodiesterases superfamily | | | 40.235 | - | - |  | - |  |
| Tc00.1047053506207.30 | lipid binding/ START domain | | | 40.380 | SKL | 360-363 |  | - |  |
| Tc00.1047053510857.10 | carn-acyltransf superfamily/ choline/carnitine o-acyltransferase | | | 78.132 | PKL | 683-685 |  | - |  |
| Tc00.1047053511071.100 | catalytic domain of phosphoinositide-specific phospholipase C-like phosphodiesterases superfamily | | | 38.879 | - | - |  | - |  |
| **Sterol synthesis** |  | | |  |  |  |  |  |  |
| Tc00.1047053506219.40 | 17-beta-hydroxysteroid dehydrogenases (17beta-HSDs) types -1,-3, and -12, -like, classical (c) SDRs | | | 34.354 | - | - |  | - |  |
| **Amino-acid metabolism** | | |  |  |  |  |  |  |  |
| Tc00.1047053508177.90 | saccharopine dehydrogenase | | | 43.453 | - | - |  | - |  |
| Tc00.1047053511691.39 | saccharopine dehydrogenase/ NAD(P)-binding Rossmann-fold domains | | | 37.396 | - | - |  | - |  |
| Tc00.1047053508821.40 | saccharopine dehydrogenase/ NAD(P)-binding Rossmann-fold domains | | | 43.242 | - | - |  | - |  |
| Tc00.1047053508177.90 | saccharopine dehydrogenase, putative *Leishmania panamensis* (96% Qc/ 41% I) | | | 47.312 | - | - |  | - |  |
| Tc00.1047053508693.50 | amidinotransferase | | | 43.565 | RFFGSKKRV | 10-18 |  | - |  |
| **Others** |  | | |  |  |  |  |  |  |
| Tc00.1047053511303.150 | phosphoribulokinase/uridine kinase family, putative *Bodo saltans* (94% Qc/ 40% I) | | | 28.005 | SSM | 248-250 |  | - |  |
| Tc00.1047053511761.60 | phosphoribulokinase/uridine kinase family, putative *Bodo saltans* (94% Qc/ 40% I) | | | 27.929 | - | - |  | - |  |
| Tc00.1047053510285.80 | chromosome segregation ATPase/ chromosome segregation protein SMC region | | | 98.933 | - | - |  | - |  |
| Tc00.1047053508501.250 | UMP-CMP kinase, mitochondrial, putative | | | 29.667 | - | - |  | - |  |
| Tc00.1047053511585.200 | protein of unknown function/ DUF3128 | | | 44.418 | - | - |  | - |  |
| Tc00.1047053511367.290 | flagellar associated protein *Trypanosoma grayi* (84% Qc/ 99% I)/ DUF667 | | | 28.365 | - | - |  | - |  |
| Tc00.1047053504147.10 | phosphoribosylpyrophosphate synthetase *Trypanosoma cruzi* Dm28c (100% Qc/ 98% I) | | | 99.813 | - | - |  | - |  |
| Tc00.1047053507949.250 | protein of unknown function/ DUF2946 | | | 39.954 | - | - |  | - |  |
| Tc00.1047053506195.290 | diguanylate cyclase *Serratia plymuthica* (95% Qc/ 63% I)/ DUF1479 | | | 48.797 | SNKL | 421-424 |  | - |  |
| Tc00.1047053510797.30 | protein of unknown function/ DUF1126 | | | 87.561 | - | - |  | - |  |
| Tc00.1047053510089.110 | mitochondrial RNA binding complex 1 subunit, putative | | | 73.113 | - | - |  | - |  |
| Tc00.1047053511555.110 | domain of unknown function/ DUF4486 | | | 429.022 | - | - |  | - |  |
| Tc00.1047053509683.10 | quinoprotein alcohol dehydrogenase-like domains | | | 326.277 | - | - |  | - |  |
| Tc00.1047053504087.20 | Quinoprotein alcohol dehydrogenase-like domains | | | 325.494 | - | - |  | - |  |
| Tc00.1047053504153.120 | frag 1/DRAM/Sfk 1 family/DNase I-like | | | 148.633 | - | - |  | - |  |
| Tc00.1047053509777.130 | inositol phosphosphingolipid phospholipase C-Like, putative (ISCL) | | | 67.360 | - | - |  | - |  |
| Tc00.1047053507641.250 | PLAC8 family | | | 13.861 | - | - |  | - |  |
| Tc00.1047053509107.9 | the Major Facilitator Superfamily (MFS) is a large and diverse group of secondary transporters that includes uniporters, symporters, and antiporters. MFS proteins facilitate the transport across cytoplasmic or internal membranes of a variety of substrates | | | 65.356 | - | - |  | - |  |
| Tc00.1047053506587.70 | hypothetical protein, conserved | | | 31.586 | - | - |  | - |  |
| Tc00.1047053507049.160 | hypothetical protein, conserved | | | 73.762 | SKL | 647-649 |  | - |  |
| Tc00.1047053506357.190 | hypothetical protein, conserved | | | 71.330 | AKL | 629-631 |  | - |  |
| Tc00.1047053508241.130 | mRNA processing protein, putative | | | 121.676 | KLHRLLQRI | 4-12 |  | - |  |
| Tc00.1047053511815.40 | guide RNA associated protein, GAP2, putative | | | 52.890 | AKL | 469-471 |  | - |  |
| Tc00.1047053506297.220 | hypothetical protein, conserved | | | 27.638 | SSL | 266-268 |  | - |  |
| Tc00.1047053508891.40 | hypothetical protein, conserved | | | 71.333 | AKL | 629-631 |  | - |  |
| Tc00.1047053506147.120 | hypothetical protein, conserved | | | 28.889 | SKRLI | 247-251 |  | - |  |
| Tc00.1047053506977.60 | MICOS complex subunit MIC10-1, putative | | | 12.440 | RVSTVASEKV | 4-13 |  | - |  |
| Tc00.1047053508387.60 | protein of unknown function/transmembrane domain (151-173) | | | 25.872 | SKAA | 237-240 |  | - |  |
| Tc00.1047053510729.230 | hypothetical protein, conserved | | | 29.620 | SRLQF | 272-276 |  | - |  |
| Tc00.1047053506925.200 | hypothetical protein, conserved | | | 89.530 | - | - |  | - |  |
| Tc00.1047053507009.20 | protein of unknown function/ Dpy-30 motif | | | 27.547 | - | - |  | - |  |
| Tc00.1047053508989.110 | insulin/insulin-like growth factor/relaxin family; insulin family of proteins. | | | 19.364 | QIHQANLKA | 7-15 |  | - |  |
| Tc00.1047053509109.30 | haloacid dehalogenase superfamily, subfamily IA/ phosphotransferase enzyme family/ PKc like catalytic domain of the protein kinase superfamily /ATP binding site | | | 95.337 | SRL | 824-826 |  | - |  |
| Tc00.1047053506399.60 | ubiquitin associated domain (the domain is a commonly occurring sequence motif in some members of the ubiquitination pathway, UV excision repair proteins, and certain protein kinases). | | | 40.474 | SRL | 358-360 |  | - |  |
| Tc00.1047053404843.20 | tetraspanin family | | | 31.654 | YRV | 276-278 |  | - |  |
| Tc00.1047053511037.20 | L-2-hydroxyglutarate dehydrogenase, mitochondrial, putative | | | 58.073 | KIFTWAVRL | 4-12 |  | - |  |
| Tc00.1047053509243.30 | leucine-rich repeats (LRRs), ribonuclease inhibitor (RI)-like subfamily. | | | 69.750 | KVDATLARF | 6-14 |  | - |  |
| Tc00.1047053509733.110 | Zf-RanBP2 /RanBP2-type zinc finger | | | 61.282 | - | - |  | - |  |
| Tc00.1047053508461.210 | dimerization-anchoring domain of cAMP-dependent protein kinase regulatory subunit/EF-hand | | | 30.129 | - | - |  | - |  |
| Tc00.1047053503811.60 | elongation factor Ts, mitochondrial, putative (EF-Ts) | | | 30.267 | RLRANVNKV | 6-14 |  | - |  |
| Tc00.1047053508737.30 | kinetoplast polyadenylation/uridylation factor 1 | | | 113.729 | SDI | 992-994 |  | - |  |
| Tc00.1047053509767.130 | LicD family | | | 40.505 | - | - |  | - |  |
| Tc00.1047053509589.40 | type VI protein secretion system component VasK | | | 152.658 | RVRRVSGRV | 2-10 |  | - |  |
| Tc00.1047053508089.50 | pyridine nucleotide-disulphide oxidoreductase/FAD/NAD(P) binding domain | | | 57.406 | SHL | 503-505 |  | - |  |
| Tc00.1047053510101.380 | macro domain, EF-hand superfamily. | | | 108.630 | RVRVAQRRL | 10-20 |  | - |  |
| Tc00.1047053508737.110 | chromosome segregation protein SMC | | | 108.817 | PSL | 935-937 |  | - |  |
| Tc00.1047053509885.50 | chromosome segregation protein | | | 112.072 | YKV | 975-977 |  | - |  |
| Tc00.1047053509099.160 | adenylate kinase putative | | | 129.468 | VSL | 1123-1125 |  | - |  |
| Tc00.1047053510879.30 | RNA-binding domain of signal recognition particle subunit 68 | | | 66.257 | - | - |  | - |  |
| Tc00.1047053511003.30 | mitochondrial RNA binding complex 1 subunit, putative (REH2) | | | 245.940 | - | - |  | - |  |
| Tc00.1047053507641.160 | Major Vault Protein repeat | | | 97.337 | SSA | 858-860 |  | - |  |
| Tc00.1047053510941.6 | protein of unknown function/ DDRGK motif | | | 36.534 | - | - |  | - |  |
| Tc00.1047053508177.10 | protein of unknown function/ WD40 motif | | | 69.037 | - | - |  | - |  |
| Tc00.1047053503953.10 | protein of unknown function/ Dpy-30 motif | | | 60.192 | - | - |  | - |  |
| Tc00.1047053510089.190 | protein of unknown function/ WD40 motif | | | 114.289 | - | - |  | - |  |
| Tc00.1047053511045.50 | protein of unknown function/ Dpy-30 motif | | | 28.957 | - | - |  | - |  |
| Tc00.1047053506957.120 | protein of unknown function/ WD40 motif | | | 235.908 | - | - |  | - |  |
| Tc00.1047053506511.20 | hypothetical protein, conserved | | | 24.833 | - | - |  | - |  |
| Tc00.1047053509789.59 | AAA domain containing protein, putative | | | 206.011 | - | - |  | - |  |

**Table SIII. Proteins involved in the metabolism of sugars detected by mass spectrometry analysis of *T. cruzi* glycosomes.** Identified PTS1 and PTS2 motifs and their location in the proteins are indicated.

| **Accession Number** | **Description** | **MW** | | **PTS** | **Position** |
| --- | --- | --- | --- | --- | --- |
| **Glycolytic metabolism** | |  | |  |  |
| Tc00.1047053510121.20 | hexokinase | | 52.676 | RLNNLLQHI | 1-12 |
| Tc00.1047053510187.100 | glucokinase 1 | | 42.010 | AQL | 374-376 |
| Tc00.1047053510889.221 | glucose-6-phosphate isomerase | | 50.395 | SHL | 606-608 |
| Tc00.1047053506529.508 | glucose-6-phosphate isomerase | | 68.514 | SHL | 606-608 |
| Tc00.1047053508153.340 | 6-phospho-1-fructokinase | | 53.988 | SKL | 483-485 |
| Tc00.1047053510301.20 | fructose-bisphosphate aldolase | | 41.181 | RVEVLQTQL | 1-12 |
| Tc00.1047053504163.40 | fructose-bisphosphate aldolase | | 41.207 | RVEVLQTQL | 1-12 |
| Tc00.1047053508647.200 | triosephosphate isomerase | | 27.516 | --- | --- |
| Tc00.1047053506943.50 | glyceraldehyde 3-phosphate dehydrogenase | | 39.236 | ARL | 357-359 |
| Tc00.1047053511419.40 | phosphoglycerate kinase | | 44.783 | QVDVKGKRV | 11-19 |
| Tc00.1047053511419.50 | phosphoglycerate kinase | | 64.871 | --- | --- |
| Tc00.1047053504153.20 | phosphoglycerate kinase | | 100.444 | --- | --- |
| Tc00.1047053506125.30 | phosphoglycerate kinase | | 22.519 | --- | --- |
| Tc00.1047053506835.70 | PAS-domain containing phosphoglycerate kinase | | 58.408 | PRL | 525-527 |
| Tc00.1047053506945.20 | PAS-domain containing phosphoglycerate kinase | | 58.422 | PRL | 525-527 |
| Tc00.1047053511277.60 | alcohol dehydrogenase | | 42.302 | --- | --- |
| Tc00.1047053506263.20 | D-isomer specific 2-hydroxyacid dehydrogenase-protein | | 22.342 | --- | --- |
| Tc00.1047053510099.120 | D-isomer specific 2-hydroxyacid dehydrogenase-protein | | 38.771 | --- | --- |
| Tc00.1047053506263.30 | D-isomer specific 2-hydroxyacid dehydrogenase-protein | | 39.639 | --- | --- |
| Tc00.1047053507641.60 | aldehyde dehydrogenase | | 54.570 | SRI | 502-504 |
| Tc00.1047053509065.9 | aldehyde dehydrogenase (fragment) | | 37.929 | --- | --- |
|  | | | | | |
| **Gluconeogenesis** |  |  | |  |  |
| Tc00.1047053506649.70 | fructose-1,6-bisphosphatase | | 38.139 | SKL | 342-344 |
| Tc00.1047053508351.10 | fructose-1,6-bisphosphatase | | 38.103 | SKL | 342-344 |
|  |  |  | |  |  |
| **Galactose metabolism** | | | | | |
| Tc00.1047053510667.120 | galactokinase | | 52.157 | GKL | 461-463 |
| Tc00.1047053507009.40 | galactokinase | | 52.182 | GKL | 460-462 |
| Tc00.1047053508465.90 | galactokinase | | 52.269 | SNL | 466-468 |
| Tc00.1047053507001.110 | galactokinase-like protein | | 52.440 | SNL | 466-468 |
|  |  | |  |  |  |
| [**Sugar-nucleotide synthesis**](http://en.wikipedia.org/wiki/Aminosugars_metabolism) | | | | | |
| Tc00.1047053506405.10 | phosphomannomutase-like protein | | 65.414 | SNL | 583-585 |
| Tc00.1047053511531.50 | glucosamine-6-phosphate isomerase | | 31.593 | SKM | 227-279 |
| Tc00.1047053511025.50 | glucosamine-6-phosphate isomerase | | 31.593 | SKM | 277-279 |
| Tc00.1047053511717.90 | phosphomannose isomerase | | 45.938 | AHI | 406-408 |
| Tc00.1047053503677.10 | phosphomannose isomerase | | 46.060 | AHM | 406-408 |
| Tc00.1047053506341.10 | N-acetylglucosamine-6-phosphate deacetylase-like protein | | 30.823 | SHM | 274-276 |
| Tc00.1047053506507.10 | N-acetylglucosamine-6-phosphate deacetylase-like protein | | 40.716 | SHM | 362-364 |
| Tc00.1047053510259.18 | GlcNAc-PI synthesis protein (GPI3) | | 52.251 | --- | --- |
| Tc00.1047053504557.10 | UDP-Gal or UDP-GlcNAc-dependent glycosyltransferase, putative | | 43.286 | KLPTMKRKL | 5-13 |
| Tc00.1047053505163.80 | oligosaccharyl transferase subunit, putative | | 91.324 | --- | --- |
|  |  |  | |  |  |
| **Glyoxylate and dicarboxylate metabolism** | | | | | |
| Tc00.1047053505807.180 | 2-hydroxy-3-oxopropionate reductase | | 32.185 | --- | *---* |
|  |  | |  |  |  |
| **Enzymes of pathways auxiliary to glycolysis/gluconeogenesis** | | | | | |
| Tc00.1047053508441.20 | phosphoenolpyruvate carboxykinase | | 59.283 | ARL | 523-525 |
| Tc00.1047053511293.69 | glycosomal malate dehydrogenase | | 34.291 | SKL | 321-323 |
| Tc00.1047053509879.40 | fumarate hydratase | | 61.382 | KYVPLIPHV | 18-26 |
| Tc00.1047053510215.10 | NADH-dependent fumarate reductase | | 125.687 | SKM | 1140-1142 |
| Tc00.1047053503849.80 | NADH-dependent fumarate reductase | | 53.669 | SKM | 483-485 |
| Tc00.1047053503849.60 | NADH-dependent fumarate reductase | | 85.951 | RVSHITTQF | 10-18 |
| Tc00.1047053503467.9 | NADH-dependent fumarate reductase | | 64.593 | --- | --- |
| Tc00.1047053508535.10 | NADH-dependent fumarate reductase | | 133.326 | --- | *---* |
| Tc00.1047053506297.190 | pyruvate phosphate dikinase | | 101.623 | AKL | 911-943 |
| Tc00.1047053507883.80 | adenylate kinase | | 24.865 | GLK | 249-251 |
| Tc00.1047053509733.180 | adenylate kinase | | 24.296 | CKL | 212-214 |
| Tc00.1047053503479.30 | adenylate kinase | | 29.437 | --- | --- |
| Tc00.1047053508461.400 | nucleoside diphosphate kinase | | 37.823 | KVDYYDPQA | 1-18 |
| Tc00.1047053507241.30 | arginine kinase | | 40.458 | KLEAAFAKL | 9-17 |
|  |  |  | |  |  |
| **Pentose-Phosphate Pathway** | | | | | |
| Tc00.1047053506925.480 | ribokinase | | 35.719 | ARA | 328-330 |
| Tc00.1047053508625.150 | L-ribulokinase | | 63.295 | SHL | 567-569 |
| Tc00.1047053504117.20 | sedoheptulose-1,7-bisphosphatase | | 35.248 | SKL | 318-320 |
| Tc00.1047053507889.10 | transaldolase | | 36.581 | --- | --- |
| Tc00.1047053508415.40 | ADP-ribosylation factor 1 | | 20.778 | --- | --- |
|  |  |  | |  |  |
| **Glycerol metabolism** | | | | | |
| Tc00.1047053511151.90 | glycerol-3-phosphate dehydrogenase | | 67.768 | --- | --- |
| Tc00.1047053510661.60 | glycerol kinase, glycosomal | | 57.298 | AKL | 510-512 |
| Tc00.1047053503983.20 | dihydroxyacetone kinase 1-like | | 62.760 | GKL | 587-589 |
|  |  |  | |  |  |

**Table SIV. Proteins involved in the lipid metabolism detected by mass spectrometry analysis of *T. cruzi* glycosomes.**

Identified PTS1 and PTS2 motifs and their location in the proteins are indicated.

| **Accession Number** | **Description** | **MW** | **PTS** | **Position** |
| --- | --- | --- | --- | --- |
| **Fatty-acid metabolism** | |  |  |  |
| Tc00.1047053511353.4 | choline/carnitine O-acyltransferase | 37.356 | PQF | 315-317 |
| Tc00.1047053509999.90 | carnitine/choline acetyltransferase | 67.347 | SKL | 608-610 |
| Tc00.1047053508827.40 | acyl-CoA dehydrogenase | 69.367 | --- | --- |
| Tc00.1047053507547.40 | enoyl-CoA hydratase/Enoyl-CoA isomerase/3-hydroxyacyl-CoA dehydrogenase | 88.724 | RVDTILSHV | 3-11 |
| Tc00.1047053508441.70 | enoyl-CoA hydratase/Enoyl-CoA isomerase/3-hydroxyacyl-CoA dehydrogenase | 88.548 | RVDTILSHV | 3-11 |
| Tc00.1047053509717.90 | short chain 3-hydroxyacyl-coA dehydrogenase | 35.794 | YKF | 318-320 |
| Tc00.1047053506727.100 | enoyl-CoA hydratase/isomerase family protein | 36.775 | --- | --- |
| Tc00.1047053509463.30 | 3-ketoacyl-CoA thiolase | 46.718 | ANI | 435-437 |
| Tc00.1047053504055.40 | acyltransferase | 137.072 | SKM | 1211-1213 |
| Tc00.1047053506435.270 | acyltransferase | 136.941 | SKM | 1211-1213 |
| Tc00.1047053511389.150 | thiolase protein-like protein | 48.298 | --- | --- |
| Tc00.1047053507107.40 | 3,2-trans-enoyl-CoA isomerase | 39.767 | --- | --- |
| Tc00.1047053503575.50 | fatty acyl CoA synthetase | 79.432 | --- | --- |
| Tc00.1047053494675.10 | fatty acyl CoA synthetase 2 | 60.654 | --- | --- |
| Tc00.1047053506661.20 | fatty acid elongase | 32.004 | --- | --- |
| Tc00.1047053510877.55 | acyl-CoA binding protein | 10.798 | SKL | 91-93 |
|  |  |  |  |  |
| **Ether-lipid synthesis** | | | | |
| Tc00.1047053503815.10 | alkyl-dihydroxyacetone phosphate synthase | 69.956 | SHL | 611-613 |
| **Sterol synthesis** | | | | |
| Tc00.1047053511903.40 | HMG-CoA-S-prok /3-hydroxy-3-methylglutaryl CoA synthase | 55.118 | --- | --- |
| Tc00.1047053506831.40 | 3-hydroxy-3-methylglutaryl-CoA reductase | 46.711 | --- | *---* |
| Tc00.1047053509237.10 | mevalonate kinase | 35.822 | AKI | 326-328 |
| Tc00.1047053436521.9 | mevalonate kinase | 39.851 | --- | --- |
| Tc00.1047053510431.10 | isopentenyl-diphosphate delta-isomerase | 19.690 | SSL | 177-179 |
| Tc00.1047053408799.19 | isopentenyl-diphosphate delta-isomerase | 20.405 | --- | --- |
| Tc00.1047053509589.20 | squalene monooxygenase | 64.497 | --- | --- |
| Tc00.1047053506297.260 | lanosterol 14-alpha-demethylase | 55.246 | --- | --- |
| Tc00.1047053510873.10 | NAD(P)-dependent steroid dehydrogenase protein (fragment) | 26.150 | --- | --- |
| Tc00.1047053505683.10 | sterol 24-C-methyltransferase | 40.998 | --- | --- |
| Tc00.1047053510329.90 | C-8 sterol isomerase | 24.323 | GKI | 216-218 |
| Tc00.1047053507709.90 | sterol C-24 reductase | 58.305 | --- | --- |
| Tc00.1047053457251.10 | 3-oxo-5-alpha-steroid 4-dehydrogenase | 29.870 | --- | --- |
|  |  |  |  |  |

**Table SV. Proteins involved in various metabolic process detected by mass spectrometry analysis of *T. cruzi* glycosomes.**

This table lists enzymes of several processes not covered by tables SII – SIV. Identified PTS1 and PTS2 motifs and their location in the proteins are indicated.

| **Accession Number** | **Description** | | | **MW** | **PTS** | **Position** |  |
| --- | --- | --- | --- | --- | --- | --- | --- |
| **Purine and pyrimidine synthesis** | | | |  |  |  |  |
| Tc00.1047053507059.60 | orotidine-5-phosphate decarboxylase/orotate phosphoribosyltransferase | | | 50.056 | SKL | 456-458 |  |
| Tc00.1047053508373.29 | orotidine-5-phosphate decarboxylase/orotate phosphoribosyltransferase | | | 29.065 | --- | --- |  |
| Tc00.1047053506519.130 | guanosine monophosphate reductase | | | 52.961 | SKL | 489-491 |  |
| Tc00.1047053508909.20 | guanosine monophosphate reductase | | | 52.846 | SKL | 489-491 |  |
| Tc00.1047053511301.110 | inosine-5'-monophosphate dehydrogenase | | | 56.118 | SKL | 510-512 |  |
| Tc00.1047053507211.40 | inosine-5'-monophosphate dehydrogenase | | | 56.137 | SKL | 510-512 |  |
| Tc00.1047053508207.70 | adenine phosphoribosyltransferase | | | 24.474 | SHM | 223-225 |  |
| Tc00.1047053507519.150 | adenine phosphoribosyltransferase | | | 24.538 | SHM | 223-225 |  |
| Tc00.1047053507519.140 | adenine phosphoribosyltransferase | | | 26.038 | SRI | 233-235 |  |
| Tc00.1047053508207.74 | adenine phosphoribosyltransferase | | | 25.999 | SRI | 233-235 |  |
| Tc00.1047053506457.40 | hypoxanthine-guanine phosphoribosyltransferase | | | 26.000 | AHL | 229-231 |  |
| Tc00.1047053509693.80 | hypoxanthine-guanine phosphoribosyltransferase | | | 26.000 | AHL | 229-231 |  |
| Tc00.1047053509693.70 | hypoxanthine-guanine phosphoribosyltransferase | | | 28.171 | SKY | 239-241 |  |
| Tc00.1047053506457.30 | hypoxanthine-guanine phosphoribosyltransferase | | | 25.627 | SKY | 219-221 |  |
| Tc00.1047053511857.80 | AMP deaminase | | | 91.603 | SRL | 789-791 |  |
| Tc00.1047053509569.100 | nucleoside phosphorylase | | | 29.250 | HLNCKSDQL | 26-34 |  |
| Tc00.1047053504103.109 | phosphorybosylpyrophosphate synthetase | | | 99.813 | SQL | 295-297 |  |
|  |  | | |  |  |  |  |
| **Trypanothione synthesis** | | | | | | | |
| Tc00.1047053508971.40 | acetylornithine deacetylase-like | | | 43.979 | SRL | 393-395 |  |
| Tc00.1047053511899.40 | 2-amino-3-ketobutyrate coenzyme A ligase | | | 43.928 | --- | --- |  |
| Tc00.1047053509099.50 | trypanothione synthetase | | | 74.096 | --- | --- |  |
|  |  | | |  |  |  |  |
| **Antioxidant defense** | | | | | | |  |
| Tc00.1047053503899.130 | glutathione peroxidase-like protein | | | 19.812 | QILDADHQL | 17-25 |  |
| Tc00.1047053508265.10 | glutathione-S-transferase/glutaredoxin | | | 35.431 | --- | --- |  |
| Tc00.1047053507259.10 | tryparedoxin peroxidase | | | 22.788 | --- | --- |  |
| Tc00.1047053508649.5 | tryparedoxin peroxidase | | | 25.718 | --- | --- |  |
| Tc00.1047053484299.10 | trypanothione reductase | | | 54.234 | ASL | 187-189 |  |
| Tc00.1047053504507.5 | trypanothione reductase | | | 42.798 | --- | --- |  |
| Tc00.1047053509997.30 | tryparedoxin 1 | | | 16.141 | KYLPSTIKL | 6-14 |  |
| Tc00.1047053511715.10 | iron superoxide dismutase | | | 23.511 | --- | --- |  |
| Tc00.1047053508445.20 | iron superoxide dismutase | | | 23.511 | --- | --- |  |
|  | | | | | | |  |
| **Redox metabolism** | |  | |  |  |  |  |
| Tc00.1047053503873.10 | cytochrome-B5 reductase | | | 34.683 | YRF | 306-308 |  |
| Tc00.1047053508543.140 | electron transfer flavoprotein-ubiquinone oxidoreductase | | | 63.015 | AQM | 562-564 |  |
| Tc00.1047053508409.160 | thymine dioxygenase | | | 36.977 | --- | --- |  |
| Tc00.1047053505183.30 | malic enzyme | | | 62.977 | --- | --- |  |
| Tc00.1047053508647.280 | malic enzyme | | | 63.048 | --- | --- |  |
| Tc00.1047053505183.20 | malic enzyme | | | 63.975 | --- | --- |  |
|  | | | | | | |  |
| **Amino-acid metabolism** | | |  |  |  |  |  |
| Tc00.1047053506247.220 | histidine ammonia-lyase | | | 58.559 | SKM | 532-534 |  |
| Tc00.1047053504045.110 | urocanate hydratase | | | 75.406 | --- | --- |  |
| Tc00.1047053509137.30 | imidazolonepropionase | | | 47.274 | QVVCGGERY | 16-24 |  |
| Tc00.1047053506411.30 | proline dehydrogenase | | | 65.326 | VKA | 564-566 |  |
| Tc00.1047053509207.90 | pyrroline-5-carboxylate reductase | | | 28.997 | ANLN | 266-269 |  |
| Tc00.1047053510943.50 | delta-1-pyrroline-5-carboxylate dehydrogenase | | | 63.008 | --- | --- |  |
| Tc00.1047053503577.9 | delta-1-pyrroline-5-carboxylate dehydrogenase | | | 29.784 | --- | --- |  |
| Tc00.1047053507923.10 | L-threonine 3-dehydrogenase | | | 37.223 | PSL | 330-332 |  |
| Tc00.1047053506681.70 | threonine dehydratase-like | | | 27.031 | ARL | 244-246 |  |
| Tc00.1047053510889.140 | alanine aminotransferase | | | 55.497 | --- | --- |  |
| Tc00.1047053506529.430 | alanine aminotransferase | | | 55.585 | --- | --- |  |
| Tc00.1047053508111.30 | glutamate dehydrogenase | | | 45.427 | --- | --- |  |
| Tc00.1047053510187.30 | tyrosine aminotransferase | | | 46.694 | --- | --- |  |
| Tc00.1047053506629.220 | isovaleryl-coA dehydrogenase | | | 45.401 | --- | --- |  |
| Tc00.1047053506853.50 | 2-oxoisovalerate dehydrogenase alpha subunit | | | 49.210 | YNM | 429-431 |  |
| Tc00.1047053506851.20 | choline dehydrogenase | | | 59.446 | --- | --- |  |
|  | | | | | | |  |
| **TCA cycle** | | | | | | |  |
| Tc00.1047053510351.90 | dihydrolipoamide branched chain transacylase | | | 47.760 | RIALWPHRI | 3-11 |  |
| Tc00.1047053507601.70 | dihydrolipoamide branched chain transacylase | | | 47.611 | RIALWPHRI | 3-11 |  |
| Tc00.1047053506925.319 | isocitrate dehydrogenase | | | 47.114 | SKI | 411-413 |  |
| Tc00.1047053510717.30 | 2-oxoglutarate dehydrogenase E1 component | | | 112.896 | RVPFVNCRV | 13-21 |  |
| Tc00.1047053511909.40 | succinate dehydrogenase (ubiquinone) flavoprotein subunit, mitochondrial (SDH1-1) | | | 67.774 | RFSPKLSKA | 8-16 |  |
|  |  | | |  |  |  |  |
| **Proteases** | | | | | | |  |
| Tc00.1047053508999.220 | calpain-like cysteine peptidase | | | 83.262 | VRA | 727-729 |  |
| Tc00.1047053506563.200 | calpain cysteine peptidase | | | 83.432 | VRA | 727-729 |  |
| Tc00.1047053504153.160 | metallo-peptidase | | | 58.197 | RVFTKLYRY | 9-17 |  |
| Tc00.1047053506513.110 | peptidase T | | | 46.958 | PRL | 417-419 |  |
| Tc00.1047053507305.30 | casein kinase | | | 38.313 | --- | --- |  |
| Tc00.1047053507689.30 | glutamamyl carboxypeptidase | | | 44.222 | AHL | 394-396 |  |
| Tc00.1047053507689.40 | glutamamyl carboxypeptidase | | | 44.090 | AHL | 394-396 |  |
| Tc00.1047053507689.50 | glutamamyl carboxypeptidase | | | 28.912 | AHL | 257-259 |  |
| Tc00.1047053507657.20 | glutamamyl carboxypeptidase | | | 44.212 | ARL | 394-396 |  |
| Tc00.1047053511391.120 | glutamamyl carboxypeptidase | | | 44.362 | PHL | 392-394 |  |
| Tc00.1047053510837.20 | glutamamyl carboxypeptidase | | | 44.191 | AHL | 394-396 |  |
|  |  | | |  |  |  |  |

**Table SVI. Transporters/integral membrane proteins detected by mass analysis of *T. cruzi* glycosomes.**

Identified PTS1 and PTS2 motifs and their location in the proteins are indicated.

| **Accession Number** | **Description** | | **MW** | **PTS** | **Position** | |
| --- | --- | --- | --- | --- | --- | --- |
| **Transporter/integral membrane protein** | | |  |  |  |  |
| Tc00.1047053508479.230 | PEX2 | | 37.817 | --- | --- |  |
| Tc00.1047053503809.20 | peroxisome assembly protein (PEX12) | | 45.691 | --- | --- |  |
| Tc00.1047053508479.190 | peroxisome assembly protein (PEX10) | | 34.245 | TSA | 298-300 |  |
| Tc00.1047053511145.40 | peroxin 14 | | 40.541 | --- | --- |  |
| Tc00.1047053507009.10 | GIM5A protein | | 25.022 | --- | --- |  |
| Tc00.1047053508613.30 | peroxisomal membrane protein 4 | | 25.151 | TSF | 218-220 |  |
| Tc00.1047053510043.40 | peroxin 16 | | 53.844 | --- | --- |  |
| Tc00.1047053504005.40 | peroxin 11 | | 38.020 | --- | --- |  |
| Tc00.1047053503811.40 | peroxisomal targeting signal type 2 receptor (PEX7) | | 28.521 | --- | --- |  |
| Tc00.1047053509877.50 | glycosomal membrane protein (PEX11) | | 24.634 | --- | --- |  |
| Tc00.1047053511017.50 | glycosomal membrane protein (PEX11) | | 24.095 | --- | --- |  |
| Tc00.1047053503833.30 | putative PEX13 | | 42.032 | TKL | 384-386 |  |
| Tc00.1047053506249.70 | ABC transporter | | 75.258 | TKF | 663-665 |  |
| Tc00.1047053506925.530 | ABC transporter (GAT3) | | 74.938 | --- | --- |  |
| Tc00.1047053510431.150 | ABC transporter (GAT2) | | 71.338 | --- | --- |  |
| Tc00.1047053506579.10 | ABC transporter | | 78.772 | --- | --- |  |
| Tc00.1047053506989.30 | ABC transporter | | 209.321 | QFRAFLWKV | 8-16 |  |
| Tc00.1047053507241.39 | ABC transporter | | 32.096 | --- | --- |  |
| Tc00.1047053508231.190 | ABC transporter | | 75.300 | TKF | 663-665 |  |
| Tc00.1047053504867.20 | ABC transporter | | 83.341 | --- | --- |  |
| Tc00.1047053511537.8 | ABC transporter of the mitochondrion | | 35.501 | --- | --- |  |
| Tc00.1047053506025.4 | membrane-bound acid phosphatase | | 33.957 | --- | --- |  |
| Tc00.1047053511209.69 | membrane-bound acid phosphatase | | 16.244 | --- | --- |  |
| Tc00.1047053508827.10 | membrane-bound acid phosphatase | | 24.508 | --- | *---* |  |
| Tc00.1047053510649.24 | membrane associated protein (partial) | | 242.007 | --- | --- |  |
| Tc00.1047053508551.39 | hexose transporter (partial) | | 43.392 | --- | --- |  |
| Tc00.1047053506355.10 | hexose transporter | | 59.621 | --- | --- |  |
| Tc00.1047053511583.40 | tricarboxylate carrier | | 36.228 | --- | --- |  |
| Tc00.1047053510581.20 | receptor-type adenylate cyclase | | 54.763 | --- | --- |  |
| Tc00.1047053508415.40 | ADP-ribosylation factor 1 | | 20.778 | --- | --- |  |
| Tc00.1047053506657.40 | ADP/ATP translocase | | 38.578 | --- | --- |  |
| Tc00.1047053506773.90 | amino acid permease/transporter | | 51.055 | --- | --- |  |
| Tc00.1047053506153.10 | amino acid transporter | | 49.547 | --- | --- |  |
| Tc00.1047053507585.10 | amino acid transporter | | 52.839 | --- | --- |  |
| Tc00.1047053509197.20 | cation transporter | | 39.493 | --- | --- |  |
| Tc00.1047053508699.130 | cation transporter | | 39.922 | --- | --- |  |
|  | | | | | |  |
| **Other proteins with a PTS** | |  | |  |  |  |
| Tc00.1047053506181.104 | acetyltransferase-like protein | | 19.237 | SKM | 165-167 |  |
| Tc00.1047053506999.90 | Rieske iron-sulfur protein (RISP), mitochondrial precursor | | 3.107 | GKL | 295-297 |  |
| Tc00.1047053510759.120 | Rieske iron-sulfur protein (RISP), mitochondrial precursor | | 34.061 | GKL | 295-297 |  |
| Tc00.1047053503419.30 | thiol-dependent reductase 1 | | 51.049 | RLCPFCQRV | 10-18 |  |
| Tc00.1047053509105.70 | thiol-dependent reductase 1 | | 51.055 | RICPFCQRV | 10-18 |  |
| Tc00.1047053511287.49 | aldo-keto reductase (AKR) | | 32.775 | QLGLGVWRA | 18-26 |  |
| Tc00.1047053506295.130 | prohibitin | | 33.101 | GRL | 304-306 |  |
| Tc00.1047053506989.190 | heat shock protein 90 | | 86.969 | GDL | 760-762 |  |
| Tc00.1047053509353.40 | PTP1-interacting protein | | 39.953 | SRL | 340-342 |  |
| Tc00.1047053506843.40 | retrotransposon hot spot protein | | 111.937 | NKI | 972-974 |  |
| Tc00.1047053503399.10 | hydrolase-like protein | | 44.670 | SRL | 404-406 |  |
| Tc00.1047053506829.80 | nicotinamidase | | 24.638 | ASL | 221-223 |  |
| Tc00.1047053505945.90 | folate/pteridine transporter | | 21.195 | VLK | 286-288 |  |
| Tc00.1047053510381.60 | proliferator-activated receptor-interacting protein (PRIP) interacting protein (PIMT) | | 22.784 | KYYGQRHRL | 16-24 |  |
| Tc00.1047053510955.40 | axoneme central apparatus protein | | 56.768 | QVFEEYQRA | 8-16 |  |
| Tc00.1047053509105.90 | MRB1-associated protein | | 34.692 | PQL | 329-331 |  |
